# Continuous Flow Biocatalytic Reductive Amination by Co‐Entrapping Dehydrogenases with Agarose Gel in a 3D‐Printed Mould Reactor

**DOI:** 10.1002/cbic.202200549

**Published:** 2022-10-26

**Authors:** Federico Croci, Jan Vilím, Theodora Adamopoulou, Vasilis Tseliou, Peter J. Schoenmakers, Tanja Knaus, Francesco G. Mutti

**Affiliations:** ^1^ van' t Hoff Institute for Molecular Sciences HIMS-Biocat & Analytical Chemistry University of Amsterdam Science Park 904 1098 XH Amsterdam The Netherlands

**Keywords:** 3D-printing, amine dehydrogenases, biocatalysis, flow chemistry, reductive amination

## Abstract

Herein, we show how the merge of biocatalysis with flow chemistry aided by 3D‐printing technologies can facilitate organic synthesis. This concept was exemplified for the reductive amination of benzaldehyde catalysed by co‐immobilised amine dehydrogenase and formate dehydrogenase in a continuous flow micro‐reactor. For this purpose, we investigated enzyme co‐immobilisation by covalent binding, or ion‐affinity binding, or entrapment. Entrapment in an agarose hydrogel turned out to be the most promising solution for this biocatalytic reaction. Therefore, we developed a scalable and customisable approach whereby an agarose hydrogel containing the co‐entrapped dehydrogenases was cast in a 3D‐printed mould. The reactor was applied to the reductive amination of benzaldehyde in continuous flow over 120 h and afforded 47 % analytical yield and a space‐time yield of 7.4 g L day^−1^ using 0.03 mol% biocatalysts loading. This work also exemplifies how rapid prototyping of enzymatic reactions in flow can be achieved through 3D‐printing technology.

## Graphical abstract

**A customisable** approach, whereby an agarose hydrogel containing co‐entrapped dehydrogenases was cast in a 3D‐printed mould, was developed. The reactor was applied to the reductive amination of benzaldehyde in continuous flow over 120 h (space‐time‐yield 7.4 g L^−1^ day^−1^, 0.03 mol% biocatalysts loading). This work also shows how 3D‐printing technology can accelerate prototyping of enzymatic reactions in flow.

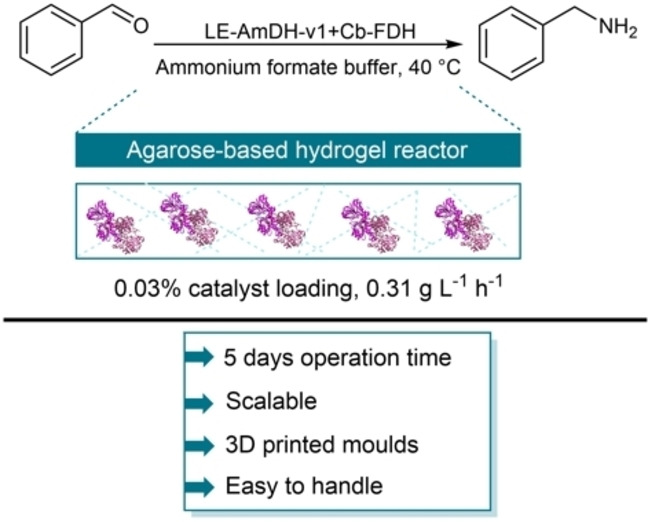

## Introduction

The International Union of Pure and Applied Chemistry (IUPAC) recently identified directed evolution of enzymes and flow chemistry among the ‘ten chemical innovations that will change our world’ due to their contribution towards more sustainable manufacturing of chemicals.^[1]^ With regard to the former, the wider implementation of natural and engineered enzymes with elevated chemo‐/stereo‐selectivities and expanded substrate scope in chemical synthesis has shortened multi‐step synthesis routes and reduced waste generation.^[2]^ In the latter case, the growing impact of continuous flow chemistry is evinced by an increasing number of studies and applications in academic and industrial settings.^[3]^ It is therefore undeniable that flow chemistry combined with biocatalysis can open up new opportunities for sustainable and efficient chemical synthesis.[^3a^, ^3e^, ^4^]

**Figure 1:**
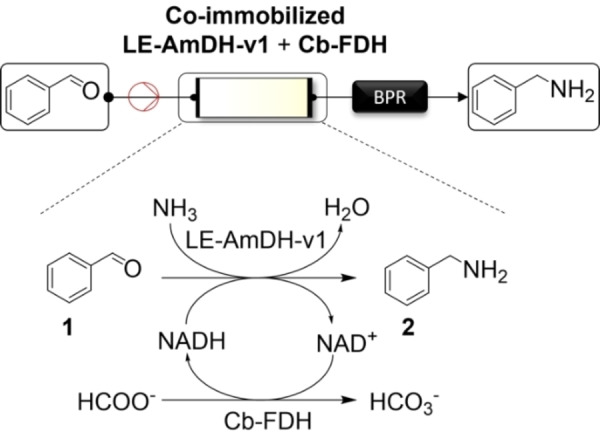
Biocatalytic flow‐system for the reductive amination using LE‐AmDH‐v1 and Cb‐FDH; BPR: back‐pressure regulator.

The development of enzyme immobilisation techniques to spatially localise the biocatalyst(s) plays a critical role in ‘flow biocatalysis’.^[5]^ Immobilisation often enables to decrease the amount of biocatalyst required to convert a certain amount of substrate (i. e. molar ratio of substrate converted vs. enzyme) due to the confinement of the enzyme in a reduced reaction volume. Furthermore, immobilisation often eases work‐up procedures, and increases the catalytic activity and lifetime of enzymes.^[6]^ Our group and others have recently focused on the biocatalytic synthesis of α‐chiral amines in flow, as these compounds are found in 40 % of the commercialised optically active drugs.^[7]^ We have also engineered novel amine dehydrogenases (AmDHs) that possess remarkable stability and catalytic performance for the synthesis of benzylic amines from related ketones.^[8]^ Herein, we report on a flow process that we developed by co‐immobilising one of our AmDH variants (LE‐AmDH‐v1) with a formate dehydrogenase (Cb‐FDH) to perform the reductive amination of benzaldehyde (**1**) as a model substrate to benzylamine (**2**; Figure 1).

## Results and Discussion

We tested different enzyme co‐immobilisation techniques to investigate the possible pros and cons of each methodology for our target enzymes and reaction. Based on our previous studies, we initially focused on the co‐immobilisation of LE‐AmDH‐v1 and Cb‐FDH via either cation‐affinity binding or covalent binding on a solid support material.[^7a^, ^7d^, ^9^] The former method is based on the interaction between cations (e. g. Fe^3+^, Co^2+^) chelated on a carrier surface and poly‐histidine tags that are genetically fused at the N‐ or C‐terminus of the recombinant enzymes.[^6d^, ^7e^, ^10^] Instead, covalent immobilisation entails the formation of covalent bonds between the reactive moieties on the carrier surface (e. g. epoxides) and the side‐chain functionalities of the enzymes (e. g. terminal moieties of l‐lysine residues).^[6c]^ For cation‐affinity binding, we tested carrier materials from different suppliers such as EziG^1^ and EziG^3^ (pre‐loaded with Fe^3+^) and Purolite (pre‐loaded with Co^2+^) beads. For covalent enzyme immobilisation, we tested support materials based on epoxide‐amine covalent attachment such as Sepabeads EC‐EP/s and Relizyme 113/s and 403/s. In all the immobilisation experiments, we have followed the procedures that are recommended by the suppliers with slight modifications. LE‐AmDH‐v1 and Cb‐FDH (5.5 : 1, molar ratio) were co‐immobilised on these carriers at comparable loadings (see Supporting Information, Table S2 and Figure S3) and applied for initial batch experiments for the reductive amination of **1** (20 mM in 750 mM NH_4_^+^/NH_3_ buffer). The highest yields were obtained using the cation‐affinity beads such as EziG^1^, Purolite and EziG^3^ (96 %, 99 % and 43 %, respectively; for details, see Supporting Information Table S3 and Figure S4). Notably the 96 % conversion was obtained with 4.4 mg of co‐immobilised biocatalysts on 100 mg of EziG^1^, whereas the 99 % conversion was obtained with 8.1 mg of co‐immobilised biocatalysts on 100 mg of Purolite. In contrast, the covalently co‐immobilised enzymes produced 2–15 % analytical yield. Notably, the 15 % and 11 % yields were obtained with Relyzime 113/s and Sepabeads EC‐EP/s that had a biocatalyst loading of 2.2 mg and 2.6 mg of enzyme per 100 mg of carrier material, respectively. Compared with the performance of cation‐affinity binding, the results obtained with Relyzime 113/s, Relyzime 403/s and Sepabeads EC‐EP/s indicate that covalent immobilisation might be less suitable for this type of reaction. In this context, the modification of the epoxide‐containing beads by creating heterofunctional chelate‐epoxy supports could enable to achieve a higher biocatalyst loading and a more stable enzyme immobilisation by merging the features of cation‐affinity binding and covalent immobilisation (i. e. “hybrid” immobilisation methodology), as described elsewhere.^[11]^

However, polymer‐coated cation‐affinity beads such as EziG^3^ exhibited another issue that was the adsorption of aldehyde **1** when tested for reaction in flow (see Supporting Information, Table S4). Notably, this issue did not occur with ketones such as acetophenone (data not shown). Therefore, we conducted extensive studies in batch using hydrophilic EziG^1^ as a carrier material (i. e. functionalised glass beads), which led to full biocatalysts co‐immobilisation at 15 % w w^−1^ loading (for details, see Supporting Information section 3.4). Catalytic activity was retained, and reductive amination of **1** (10 mM in 750 mM NH_4_^+^/NH_3_ buffer, pH 8) resulted in 56 % analytical yield of **2** (see Supporting Information, Table S6). Therefore, we attempted to transfer the process to a flow system. A stainless‐steel column was filled with EziG^1^ beads (ca. 500 mg), and the cell lysate (in Tris‐HCl buffer, 50 mL, 100 mM, pH 7.8) containing LE‐AmDH‐v1 and Cb‐FDH (total ca. 50 mg, 10 % w w^−1^ loading) was flowed through the column (0.2 ml min^−1^), followed by static incubation (30 min) at room temperature. Electrophoretic analysis of the flow‐through showed that nearly quantitative immobilisation was obtained. However, we observed partial enzyme leaching when the reaction buffer (NH_4_^+^/NH_3_ 750 mM, pH 8) containing **1** was flowed through the column (see Supporting Information, Figure S6). Notably, ca. 35 % conversion (3.5 mM product **2** concentration) was obtained in some of the collected fractions despite the biocatalyst leaching (see Supporting Information, Table S7). We verified that the leaching was due to the high concentration of the requisite NH_4_^+^/NH_3_ species for the reductive amination, which disrupted the ion‐affinity interactions between the enzymes and carrier material during operation in flow. Apparently, this issue is significantly more severe in flow than in batch.[^7f^, ^9^] In a concomitant work by Bommarius’ group, another AmDH and another FDH were successfully co‐immobilised onto another type of cation‐affinity carrier material (Bio‐Rad Nuvia IMAC).^[7g]^ However, the substrate of the reaction was a ketone and the carrier material was amido polymer beads with covalently bond nitriloacetic acid for the chelation of Ni^2+^ cations. Therefore, this type of beads is physicochemically like the EziG^3^ tested in this work, which showed strong adsorption of aldehyde substrate **1**. In fact, in another work by Turner's group an AmDH and a FDH were co‐immobilised onto EziG^3^ and applied for the reductive amination of a ketone.^[7f]^ The reaction could be run for 3 h, after which deactivation of the FDH occurred. In summary, there was no report in the literature about continuous flow reductive amination of aldehydes using co‐immobilised AmDH and FDH. With the aim of developing a method that is generally suitable for the reductive amination of carbonyl compounds in flow with AmDH and FDH, we decided to continue with the use of hydrophilic EziG^1^ beads that show no adsorption of aldehyde **1**. We attempted to avoid the enzyme leaching from EziG^1^ beads by implementing an additional cross‐linking step of the immobilised enzymes using glutaraldehyde (5 % v v^−1^).[^6b^, ^6d^] A glutaraldehyde solution in KPi buffer (100 mM, pH 7.6) was flowed through the column immediately after all the cell lysate had been loaded to the EziG^1^ carrier (see Supporting Information section 3.7). Although the cross‐linking precluded the leaching as envisioned, the additional step resulted in loss of enzyme activity (see Supporting Information, Table S8).

The issues encountered in the utilisation of LE‐AmDH‐v1 and Cb‐FDH co‐immobilised by either ion‐affinity or covalent methods in a packed‐bed flow microreactor for the reductive amination of an aldehyde (**1**) exemplify some of the obstacles to achieving a robust implementation of flow chemistry for enzymatic reactions. To address the incompatibility between the tested enzyme immobilisation methodologies and the development of a process for reductive amination of aldehyde **1** in flow, we focused on the entrapment of the enzymes as a possible solution. This technique consists in the mild encapsulation of the enzymes in a natural or synthetic polymer in the form of a hydrogel without any enzyme‐specific manipulation.[^6a^, ^6c^, ^6e^, ^12^] Next, we harnessed the advantages of 3D‐printing technology, which is increasingly having an impact in organic synthesis and flow chemistry.[^3e^, ^13^] For instance, Rabe's group recently showed that enzymes can be dispersed in a molten agarose solution (3 %) that can be subsequently 3D‐printed to create an ‘agarose gel reactor’ of pre‐defined geometry for utilisation in flow biocatalysis.^[14]^ The enzyme dispersion and printing process was performed at 60 °C to keep the agarose fluid and reduce its viscosity; therefore, a 3D‐printing device equipped with both a heated agarose‐container and heated tip was required. Although our selected enzymes possess sufficient thermostability (i. e. in particular LE‐AmDH‐v1 has a T_m_ over 60 °C),^[8]^ we envisaged an alternative approach that enables enzyme entrapment in the agarose gel at lower temperature (40 °C) without compromising subsequent utilisation in a flow reactor. Thus, our method enables work with mesophilic enzymes.

**Figure 2:**
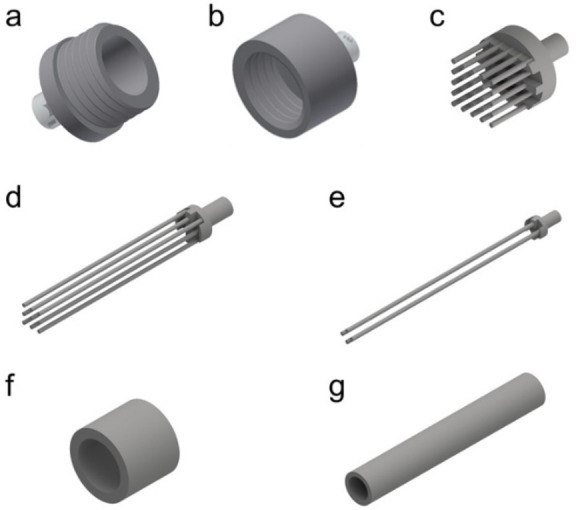
Visualisation of 3D‐printed devices: a) Type I – holder 1; b) Type I – holder 2; c) Type I – insert; d) Type II‐v1 – insert; e) Type II‐v2 – insert; f) Type I mould; g) Type II mould.

In general, the introduction of 3D‐printing in the process of enzymes’ entrapment or the manufacture of ‘biocatalytic reactors’ permits easy customisation and enables the development of more efficient processes that optimise the performance and stability of the biocatalyst(s) and reaction productivity. Accordingly, we designed and 3D‐printed a mould using a methacrylate‐based resin (Formlabs Durable) into which the agarose hydrogel containing the enzymes was subsequently cast at 40 °C (Figure 2; for details see experimental part and Supporting Information sections 4.1).

Different types of mould reactors, and variations thereof, for the agarose hydrogel were designed and produced. Table 1 reports the dimensions and Figure 2 depicts shapes and geometries of the 3D‐printed devices.

**Table 1:** List of 3D‐printed devices and their dimensions.

3D device		Height	Diameter	Wall	Pin		Reactor
Type	Part	[cm]	(inner) [cm]	thickness [cm]	n	Diameter [cm]	volume [cm^3^]
Type I	Holder 1	14.4	19.2	0.48	–	–	n.a.^[a]^
	Holder 2	15	30	0.5	–	–	n.a.^[a]^
Type I	Mould	1	1.5		–	–	1.5
	Insert	1	–		25	0.125	
Type II – v1	Mould	6	1		–	–	4.0
	Insert	6	–		9	0.125	
Type II – v2	Mould	10	1		–	–	6.0
	Insert	12	–		4	0.24	

[a] Not applicable.

**Figure 3:**
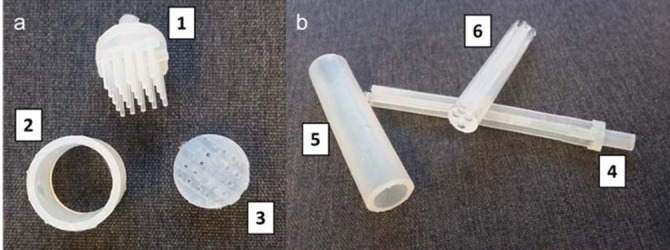
Moulds, inserts and resulting agarose‐gel reactors: a) Type I; 1=insert; 2=mould; 3=resulting hydrogel. b) Type II‐v2; 4=insert; 5=mould; 6=resulting hydrogel.

Among the different geometries, mould reactor Type II‐v2 turned out to be of most practical use (Figure 3). Type II‐v1 and v2 designs differ for the number of channels as depicted in Figures 2d and 2e, respectively. The Type II‐v1 design has nine channels, whereas the Type II‐v2 has four channels. We initially designed and tested the Type II‐v1 mould, but we found this design to be inconvenient because the cast agarose gel (containing the entrapped enzymes) had the tendency to partially break when it was going to be removed from the mould. In contrast, the Type II‐v2 (with 4 channels) facilitated the removal of the agarose gel from the mould without the risk for any break. The 3D‐printed Type II‐v2 mould was also highly resistant and durable. In fact, we could perform the whole study reported in this manuscript by using just one 3D‐printed Type II‐v2 mould and the same mould was still usable afterwards. In contrast, the Type II‐v1 proved also to be less durable because it can become distorted upon frequent usage.

The hydrogel cast from the Type II‐v2 mould (Figure 3b) also has the advantage that it can be fit into standard and commercially available empty columns while providing a good performance and robustness for the biocatalytic reaction operated in flow.

Additionally, the Type I mould (Figure 2c,f and 3a) was too short to match the reaction rate for the enzymatic reductive amination. Finally, the Type I mould was not customised to provide a cast hydrogel that could be fit into any standard column; therefore, holders had also to be 3D‐printed in this case (Figure 2a and b).

As previously described for the other immobilisation techniques, the initial activity tests with LE‐AmDH‐v1 and Cb‐FDH co‐entrapped in agarose‐gel were performed in batch (for details, see Supporting Information section 4.2 and Table S9). In general, the agarose (ca. 1 mL solution) containing LE‐AmDH‐v1 (90 μM) and Cb‐FDH (16 μM) was cast as cylindrical gel ‘discs’ with dimension of ca. 0.5 cm×0.5 cm (diameter×height) into the 3D‐printed mould. The resulting gel was sliced in bricks of similar size and placed into 2 ml vials for activity assay. The reaction was conducted again using **1** (10 mM) in an ammonium formate buffer (750 mM, pH 8). Quantitative conversion was achieved – in fact, the recovery of **2** was superior (9.0 mM) than previously reported using the immobilisation carriers; thus, the entrapment proved to be promising for overcoming adsorption of substrate and products on the heterogeneous biocatalytic system (for details, see experimental part). However, an extraction step of the agarose gel was necessary to increase the recovery of **2** from 6.9 mM to 9 mM. Next, we assayed the stability and lifetime of the entrapped enzymes in the agarose‐based hydrogel bricks. The same batch of entrapped LE‐AmDH‐v1 and Cb‐FDH was applied for seven consecutive cycles over the course of 9 days (reaction conditions: **1**, 10 mM; NAD^+^ 1 mM; HCOONH_4_ buffer, 750 mM at pH 8).

**Figure 4:**
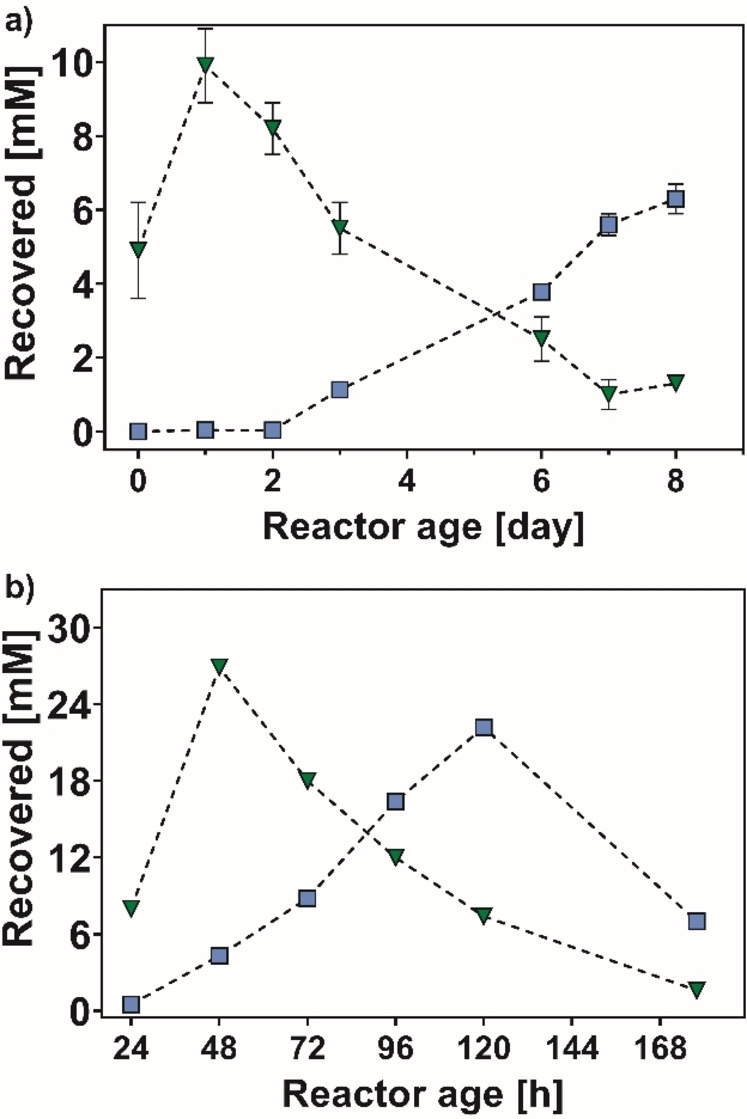
Reductive amination of **1** (blue squares) to yield **2** (green triangles) with co‐entrapped LE‐AmDH‐v1 and Cb‐FDH in agarose hydrogel: a) consecutive batch reactions (10 mM of **1**) with recycling of entrapped biocatalysts; b) reaction in flow micro‐reactor (30 mM of **1**).

Figure 4a (for details, see Supporting Information section 4.3 and Table S10) shows that the activity was retained quite well until the 4^th^ cycle and decreased from cycle 5 (equal to 6 days system age). The highest product recovery was observed in cycles 2 and 3, resulting in recovery of **2** ranging from 8 to 10 mM. The apparent lower product yield in cycle 1, when the enzymes must have the highest activity, was attributed to the tendency of the agarose gel to slow down the elution of **2** because of its diffusion through the gel. However, this amount of **2** could be recovered in the subsequent cycles as a consequence of the delayed elution. Furthermore, from the 3^rd^ cycle, the total molar amount of recovered **1** and **2** started to decrease from the expected 10 mM to ca. 7–8 mM. This fact also correlated with the decrease formation of product **2**. Therefore, we attribute this deviation from the expected mass balance to the higher volatility and the more difficult extraction of the accumulated substrate **1** after the 3^rd^ cycle compared with product **2**.

With the casting protocol in hand, we tested the LE‐AmDH‐v1 and Cb‐FDH entrapped in the agarose hydrogel in a flow system under the same reaction conditions. The use of a cast hydrogel pin‐reactor facilitates the preparation and handling of the entrapped biocatalysts and allows for a better adaptability compared with conventional spherical agarose beads. The Type II 3D‐printed mould was employed to cast the agarose solution containing the enzymes, thereby resulting in a cylindric geometry of 1×6 cm (diameter×length) with 9 narrow internal channels to increase the available superficial contact area (Type II‐v1 insert, see Figure 2d). The cast hydrogel was fit directly into an empty column (1×6 cm, diameter×length) and used to perform the reductive amination in flow (50 °C, 24 h, flow rate 0.02 ml min^−1^) at 10 mM concentration of **1**. This preliminary experiment was performed using a syringe pump (for procedures, see Supporting Information section 4.4 and Figure S9) and afforded >95 % analytical yield and full recovery of **2** upon extraction (9.5 mM). Notably, the highest previously reported yields for the reductive amination of ketones using co‐immobilised AmDH and FDH on cation‐affinity beads were 48 % and 68 %, respectively.[^7f^, ^7g^] Afterwards, we modified the geometry of the agarose hydrogel by reducing the number of internal channels from 9 to 4 (Type II‐v2 insert, see Figure 2e) to enhance its robustness, and we tested several reaction conditions (e. g. amount of co‐entrapped enzymes, substrate loading concentration, flow rate, temperature). Under such conditions (10 mM of **1**, 50 °C, flow rate 0.02 ml min^−1^, 24 h), quantitative analytical yield of **2** was obtained (for details, see Supporting Information section 4.5 and Table S11). The final flow was set to 0.02 mL min^−1^ to allow the buffer containing the substrate to enter through the pores into the agarose gels and react with the enzymes. When the flow was set too high, the substrate diffusion into the agarose gel pores was impeded and the reaction either did not occur or the conversion was greatly reduced. Subsequently, we increased the concentration of **1** from 10 mM to 30 mM to enhance the productivity while the temperature was reduced to 40 °C to minimise any possible evaporation of volatile **1** in the flow‐stream. Therefore, the optimised process was performed over the course of 120 h using 750 mM of ammonium formate buffer and 30 mM of **1** (458.4 mg, 4.31 mmol). The system afforded 47 % analytical yield of **2** (202.5 mg, 2.06 mmol), which was then isolated upon extraction in 34 % overall yield (1.47 mmol). For procedures, see experimental part and for details, see Supporting Information section 4.6 and Tables S12 and 13. Table 2 summarises the parameters of the flow process with co‐entrapped LE‐AmDH‐v1 and Cb‐FDH.

**Table 2:** Summary of flow process parameters for the reductive amination of **1** to **2**.

Parameter	Value [unit]
Reactor volume	6 ml
Total biocatalysts loading	28.1 mg (0.000636 mmol)
Flow	0.02 ml min^−1^
Reaction mixture volume	120 ml
Total loading of **1**	458.4 mg (4.31 mmol)
Analytical yield of **2**	220.5 mg (2.06 mmol)
Isolated yield of **2**	157.8 mg (1.47 mmol)
Yield per gram of catalyst	7.85 g g^−1^
Yield per mol of catalyst (TTN)	3239 mol mol^−1^
Space time yield	7.4 g L^−1^ day^−1^

Notably, we observed that the system's catalytic performance began to decrease after a maximum at 48 h (see Figure 4b and Table S12), which prevented to reach a clear steady‐state during operation. Therefore, we performed electrophoretic analysis (SDS‐PAGE) of the collected fractions to check for possible elution of any entrapped enzyme from the gel (see Supporting Information, section 4.6). Indeed, a significant amount of the NADH‐recycling enzyme Cb‐FDH eluted from the agarose gel over this period, whereas LE‐AmDH‐v1 was substantially retained (see Supporting Information Figure S10). Since both enzymes possess a similar molecular mass of their monomer (ca. 42.4 and 44.5 kDa, respectively), we attribute the different elution behaviour to the different oligomeric state of the enzymes in solution. In fact, Cb‐FDH exists in solution as a dimer,^[15]^ whereas we determined that LE‐AmDH‐v1 preferentially forms tetramers (for details, see Supporting Information section 4.8 and Figure S12 and Table S14).

To further enhance the system's catalytic performance and lifetime, future studies can consider the use of either agarose‐gel at increased concentration or a FDH with higher native molecular mass (i. e. related to the oligomeric state) or even engineer enzyme chimeras in which recombinant AmDH and FDH are genetically fused in a single polypeptide chain. We think that the latter solution is particularly promising because we could increase the enzyme size while also improving the shuttling of NAD^+^/NADH between the two dehydrogenase units. Co‐entrapment or cross‐linking of the NAD‐cofactor can be another objective for improving catalytic efficiency and reducing operation costs. However, already in this study, we could conduct the reductive amination using co‐entrapped dehydrogenases over 5 days. The implementation of the AmDH/FDH cascade in flow improved the efficiency of the reaction in terms of product formed per amount of biocatalysts (ca. 0.03 mol%), (see Table 3 and Table 2 for comparison).^[8]^

**Table 3:** Summary of the reaction parameters for the reductive amination of **1** to **2** in batch as described in analytical scale in Ref. [8].

Parameter	Value [unit]
Batch reaction volume	0.5 ml
Total biocatalyst loading	2.34 mg (0.000053 mmol)
Total loading of **1**	5.3 mg (0.050 mmol)
Analytical yield of **2**	5.3 mg (0.050 mmol)
Yield per gram of catalyst	2.26 g g^−1^
Yield per mol of catalyst (TTN)	943 mol mol^−1^
Batch reaction time	48 h
Space time yield	5.3 g L^−1^ day^−1^

## Conclusion

In summary, this work proves that the synthetic applicability of the reductive amination catalysed by amine dehydrogenases can be significantly improved by applying flow microreactors. Among the tested enzyme immobilisation techniques, entrapment was the most compatible with the enzymes and reaction conditions for the reductive amination of an aldehyde such as benzaldehyde. Therefore, we developed a scalable and customisable approach whereby an agarose hydrogel containing the entrapped dehydrogenases is cast in a 3D‐printed mould. This protocol is also applicable for mesophilic enzymes because the hydrogel‐based reactor can be prepared at lower temperature. This work also exemplifies how the incorporation of 3D‐printing technology in flow biocatalysis can generate simple and efficient solutions that can be rapidly assimilated in chemical manufacturing.

## Experimental Section

**General information**: Benzaldehyde (**1**), benzylamine (**2**), catalase, bovine albumin, agarose and CuSO_4_⋅5H_2_O were purchased from Sigma‐Aldrich. NAD^+^ was purchased from Melford Laboratories (Ipswich, UK). 2‐Propanol was obtained from Biosolve (Valkenswaard, The Netherlands). Purification of both LE‐AmDH‐v1 and Cb‐FDH was performed as previously reported.^[8]^

Supporting Information reports experimental procedures on: co‐immobilisation of LE‐AmDH‐v1 and Cb‐FDH on cation‐affinity carrier material; co‐immobilisation of LE‐AmDH‐v1 and Cb‐FDH via covalent binding on epoxide‐resins; biocatalytic reductive aminations in batch and in flow using co‐immobilised LE‐AmDH‐v1 and Cb‐FDH on these carrier materials; experiments of cross‐linking with glutaraldehyde; initial experiments of entrapment of LE‐AmDH‐v1 and Cb‐FDH in agarose‐based hydrogel; initial experiments of biocatalytic reductive aminations in batch and in flow using entrapped LE‐AmDH‐v1 and Cb‐FDH in agarose‐based hydrogel; SDS‐gel page analysis; determination of the oligomerisation state of LE‐AmDH‐v1 and Cb‐FDH; analytical procedures; GC chromatograms. All the other essential procedures are reported below.

**3D‐printed device**: 3D‐printed devices (holders and moulds) were designed using Autodesk Inventor (Autodesk, San Rafael, CA, USA). The devices were fabricated using stereolithography (i. e. building the object layer‐by‐layer in the desired shape via photo‐polymerisation of liquid resin by a scanning laser or a digital light projector) using a Form 2 3D‐printer (Formlabs, Somerville, Massachusetts, United States). 3D‐printed devices were post‐processed by sonication in 2‐propanol and compressed air to remove any uncured resin. Finally, parts were placed in a Form Cure (405 nm; Formlabs) for UV and thermal curing and cured for 60 min at 60 °C. Table 1 contains the dimensions and Figure 2 depicts shapes and geometries of the 3D‐printed devices. To enclose the Type I reactor in the holder, holder parts were equipped with straight threads #10–32 UNC (major diameter 4.83 mm, thread pitch 0.794 mm) using a hand tap. Conical ferrule seats were included in the designs to allow for a leak‐proof connection with the devices.

Reactor volume was calculated using the formula for the volume of the cylinder (V=πhr^2^) and the volume of the pins was subtracted from the total volume giving the final Equation 1.

(1) $$V=\pi {}^{*}h_{mould}{}^{*}(\frac{d_{mould}}{2}{)}^{2}-n_{pin}{}^{*}\left(\pi {}^{*}h_{mould}{}^{*}(\frac{d_{pin}}{2}{)}^{2}\right)$$

**Preparation of the agarose‐based hydrogel flow reactor**: An agarose solution (3 % w w^−1^, supplemented with NaCl 10 mM, 8 mL final volume) was prepared in Tris‐HCl buffer (100 mM, pH 7.8) and heated up with a microwave (300 W) until a clear solution was obtained. Afterwards, the solution was allowed to cool for up to 2–3 minutes and then purified LE‐AmDH‐v1 (90 μM as final concentration) and Cb‐FDH (16 μM as final concentration) were added. The resulting mixture was quickly poured into the 3D‐printed mould and allowed for cooling down and solidification. Finally, the reactor was removed from the mould, hydrated by incubation with substrate‐free reaction buffer for at least 10 min and then placed into the reactor case. After assembling the reactor, the reaction chamber was initially quickly filled with reaction buffer. Figure 3 depicts the Type I and Type II‐ v2 reactors with their respective moulds and inserts.

**Continuous‐flow reductive amination using entrapped LE‐AmDH‐v1 and Cb‐FDH on an agarose‐based hydrogel flow reactor at 30 mM substrate loading**: The agarose‐based hydrogel flow reactor (Type II‐v2) was prepared as described above. Next, it was removed from the mould and inserted into an empty HPLC‐column (1 cm×10 cm, diameter×length). The assembled reactor was subsequently mounted on a Dionex P680 HPLC pump unit (peristaltic pump) and conditioned by flowing the reaction buffer (ammonia/ammonium formate, pH 8, 750 mM). The continuous‐flow reductive amination was performed using **1** (30 mM) with 5 % of DMSO at 40 °C and 0.02 mL min^−1^ flow. To monitor the progress of the reaction, aliquots of the outflow (500 μL, each) were collected every 24 h and double‐extraction procedure was performed. Each aliquot was acidified with HCl (3 M, 70 μL) and **1** was extracted from the mixture using EtOAc (650 μL) containing 10 mM toluene as internal standard (1 min vortexing followed by 5 min centrifugation at 4 °C, 14800 rpm). Samples were dried over anhydrous MgSO_4_ and analysed by GC‐FID (see Supporting Information section 5 for analytics). Thus, analytical quantification of unreacted **1** was obtained. Next, the aqueous phases from the first extraction were basified using KOH (10 M, 150 μL) and **2** was extracted using EtOAc (650 μL) containing 10 mM toluene as internal standard, as described for the previous step. Samples were dried over anhydrous MgSO_4_ and analysed by GC‐FID (see Supporting Information section 5 for analytics). Finally, additional aliquots (30 μL, each) were analysed using SDS‐PAGE to monitor for the possible enzyme leaching from the reactor (see Supporting Information, Figure S10). In summary, 144 mL of reaction mixture were flown through the reactor resulting in the loading of 458.4 mg of **1** (4.31 mmol).

After the reaction, the combined eluted fractions were acidified with HCl (3 N, 5 mL) and the unreacted **1** was extracted with MTBE (100 mL). The aqueous solution was basified with KOH (10 M, 5 mL) and product **2** was extracted with MTBE (2×100 mL). Similarly, the agarose hydrogel reactor was sliced and washed with buffer. The resulting washing solution was first acidified with HCl (3 N, 5 mL) and unreacted **1** was extracted with MTBE (70 mL). Next, the aqueous washing phase was basified by the addition of KOH (10 M, 5 mL) and extracted using MTBE (100 mL). The obtained MTBE organic solutions were combined (approximately 300 mL in total) and dried over anhydrous MgSO_4_. After filtration and removal of the solvent under reduced pressure, a light‐yellow liquid was obtained. The purity of extracted **2** (benzylamine) was determined by GC‐FID (see Supporting Information, Figure S11). The overall process yielded 175.3 mg of **2** with 90 % purity, thus resulting in 157.8 mg (1.47 mmol) of pure **2**. However, based on the volume of collected fractions and their concentration (determined using the internal standard, see Supporting Information Table S13), the theoretical analytical yield of **2** was calculated to be 220.5 mg (2.06 mmol, 47.8 %). In summary, the analytical yield of the flow reaction for **2** was 47.8 %, while the final isolated yield of **2** (after extraction) was 34.2 %.

## Conflict of interest

The authors declare no conflict of interest.

1

## Supporting information

As a service to our authors and readers, this journal provides supporting information supplied by the authors. Such materials are peer reviewed and may be re‐organized for online delivery, but are not copy‐edited or typeset. Technical support issues arising from supporting information (other than missing files) should be addressed to the authors.

Supporting Information

## Data Availability

The data that support the findings of this study are available in the supplementary material of this article.
