# Supplementary material for: Continuous Flow Biocatalytic Reductive Amination by Co‐Entrapping Dehydrogenases with Agarose Gel in a 3D‐Printed Mould Reactor
Source: Chembiochem. 2022 Oct 26;23(22):e202200549. doi: 10.1002/cbic.202200549 (PMC9828473; doi:10.1002/cbic.202200549)
Supplement: Supplementary file 1 — Supporting Information [file CBIC-23-0-s001.pdf]

ChemBioChem

Supporting Information

Continuous Flow Biocatalytic Reductive Amination by Co-Entrapping Dehydrogenases with Agarose Gel in a 3D-Printed Mould Reactor

Federico Croci[+], Jan Vilím[+], Theodora Adamopoulou, Vasilis Tseliou, Peter J. Schoenmakers, Tanja Knaus, and Francesco G. Mutti*

Table of Contents

Table of Contents 1
Experimental Procedures 2
1. Abbreviations 2
2. General information 2
2.1 Substrates and reference compounds used in this study 2
3. Co-Immobilization of LE-AmDH-v1 and Cb-FDH on different carrier materials and reactions in batch 2
3.1. Co-Immobilization of LE-AmDH-v1 and Cb-FDH from cell free extract on EziG[3] beads and biocatalytic amination of **1** in batch 2
3.2. Co-Immobilization of LE-AmDH-v1 and Cb-FDH on other carrier materials and biocatalytic amination of **1** in batch 3
3.3. In-flow co-immobilization on EziG-Amber beads from cell free extract and initial experiments in flow 5
3.4. Co-immobilization of LE-AmDH-v1 and Cb-FDH on EziG[1] (Opal) beads at different enzyme loadings 6
3.5. Co-immobilization of LE-AmDH-v1 and Cb-FDH from cell free extract and activity test 7
3.6. In-flow co-immobilization of LE-AmDH-v1 and Cb-FDH from cell free extract 7
3.7. In-flow co-immobilization and cross-linking of dehydrogenase enzymes 8
4. Co-entrampment of LE-AmDH-v1 and Cb-FDH on an agarose-based flow reactor 9
4.1. Preparation of agarose-based hydrogel "bricks" for activity tests 9
4.2. Preliminary activity test in batch 9
4.3. Recycling test 9
4.4. In-flow reductive amination using entrapped LE-AmDH-v1 and Cb-FDH using an agarose-based hydrogel flow reactor 10
4.5. Continuous-flow reductive amination using entrapped LE-AmDH-v1 and Cb-FDH in an agarose-based hydrogel flow reactor 11
4.6. Continuous-flow reductive amination using entrapped LE-AmDH-v1 and Cb-FDH on an agarose-based hydrogel flow reactor at 30 mM substrate loading 11
4.7. Determination of process parameters from optimized experiment 13
4.8. Oligomerization of LE-AmDH-v1 14
5. Analytics 14
6. GC chromatograms 15
6.1. In-flow reductive amination using co-immobilized LE-AmDH-v1 and Cb-FDH on EziG[3](Fe- Amber) beads 15
6.2. In-flow reductive amination using co-entrapped LE-AmDH-v1 and Cb-FDH on an agarose-based hydrogel flow reactor 16
7. References 19

Experimental Procedures

1. Abbreviations

EtOAc	ethyl acetate
LE-AmDH-v1	variant 1 of the (ε-deaminating) L-Lysine dehydrogenase from *Geobacillus stearothermophilus*
Cb-FDH	formate dehydrogenase from *Candida boidinii*
CFE	cell free extract

2. General information

2.1 Substrates and reference compounds used in this study

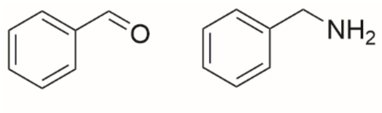

1 **2**

Fig. S1. Chemical compounds used in the study

Materials and suppliers are reported in the main manuscript, experimental part.

3. Co-Immobilization of LE-AmDH-v1 and Cb-FDH on different carrier materials and reactions in batch

*3.1. Co-Immobilization of LE-AmDH-v1 and Cb-FDH from cell free extract on EziG³ beads and biocatalytic amination of **1** in batch*

Note: The initial experiments for the co-immobilization of LE-AmDH-v1 and Cb-FDH were performed using EziG³ (Fe-Amber) cation-affinity beads. Analytical yields for initial batch reactions for the conversion of benzaldehyde (**1**) to benzylamine (**2**) at different substrate loading (10–20 mM) and buffer concentrations (375–750 mM) are reported in Table S1 and Fig. S2. Further experiments with co-immobilized LE-AmDH-v1 and Cb-FDH on the other carrier materials were conducted at 20 mM substrate concentration in 750 mM ammonium/ammonia buffer (section 3.2; Fig. S4).

The bacterial cell pellet (1 g) contaninig overexpressed LE-AmDH-v1 or Cb-FDH was re-suspended in 0.9% Tris-HCl (100 mM, pH 7.8, 5 ml) and subsequently sonicated (5 min on time, 10 s on, 10 s off, 45% amp). Afterwards, samples were centrifuged (10 min, 4 °C, 14800 rpm) and the supernatant was filtered (0.45 µm filter pores).

Co-immobilization of LE-AmDH-v1 and Cb-FDH on EziG metal-ion affinity beads was carried out as follows. Cell free extracts containing a mixture of overexpressed LE-AmDH-v1 and FDH (20 % enzymes loading w w^{-1} compared with carrier material) were added to a 15 ml falcon tube containing 200 mg of EziG³ (Fe-Amber) carrier material. The mixture was shaken on an orbital shaker at 120 rpm for 3 h at 4 °C. Small aliquots from the aqueous phase (20 µl) were taken before and after the incubation period; thus, the outcome of the immobilization was checked by analyzing the aliquots through SDS-PAGE. Finally, the immobilized enzyme was left to sediment, the buffer was removed by pipetting, and the immobilized enzyme was used directly in biotransformations.

Reaction conditions for reductive amination of benzaldehyde (1): 1 ml final volume in 2 ml Eppendorf tubes; ammonium formate buffer (375 or 750 mM, pH 8); NAD^+ (1 mM); **1** (10 or 20 mM) as substrate. Co-immobilized LE-AmDH-v1 and Cb-FDH were prepared as reported above (ca. 25 mg of total mass: enzymes on beads, for each sample). The biotransformations were incubated at 170 rpm and 30 °C in an orbital shaker for 24 h. Next, 500 µl of the reaction mixture was stopped with 70 µl of 10 M KOH, extracted once with 650 µl of EtOAc containing 10 mM toluene as internal standard (1 min vortexing + 5 min

centrifugation, 4 °C, 14800 rpm) and dried over $MgSO_4$. Analytical yields (Table S1) were determined by GC-FID (see paragraph Analytics) using toluene as internal standard. Reactions were performed in duplicate.

Table S1. Reductive amination of benzaldehyde using co-immobilized LE-AmDH-v1 and Cb-FDH. Conditions: ammonia/ammonium formate buffer (375 and 750 mM, pH 8), NAD^+ 1 mM and substrate (**1**) 10 and 20 mM; co-immobilized LE-AmDH-v1 and Cb-FDH on EziG[3] (Fe-Amber) beads, ca. 25 mg (total mass, each sample). t = 22 h, T = 30 °C, 170 rpm.

Substrate Loading [mM]	Buffer Concentration [mM]	Analytical Yield [%]
10	375	12 ± 1
20	375	22 ± 4
10	750	38 ± 5
20	750	43 ± 7

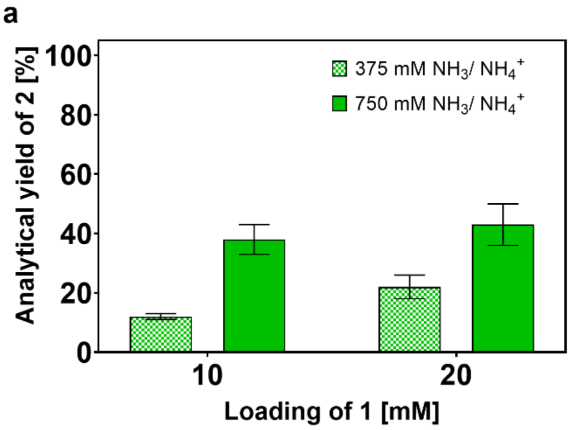

Fig. S2. Activity of LE-AmDH-v1 plus Cb-FDH co-immobilized on: EziG[3] in presence of different concentrations of ammonium/ammonia species (375–750 mM) and substrate **1** (10–20 mM).

3.2. Co-Immobilization of LE-AmDH-v1 and Cb-FDH on other carrier materials and biocatalytic amination of ***1*** *in batch*

Immobilization on EziG[1] beads. 100 mg of EziG[1] (Opal) beads pre-loaded with Fe^{3+} were poured in a 15 ml Falcon tube, cooled down in an ice bath and suspended in the immobilization buffer (Tris-HCl, 10 ml, 100 mM, pH 7.8). Purified LE-AmDH-v1 (335 µl from a 572 µM stock solution equal to 192 nmol, ca. 8.5 mg) and Cb-FDH (26 µl from a 1.33 mM stock solution equal to 35 nM, ca. 1.5 mg) were added to the suspension (total enzyme ca. 10 mg, equal to 10% w w^{-1}, enzyme loading to support material) and the mixture was shaken on an orbital shaker (120 rpm) for 3 h at 4 °C. Small aliquots from the aqueous phase (30 µl) were taken before and after the immobilization procedure; their concentrations were determined using Bradford analysis and visualized via SDS-PAGE. The carrier bearing the immobilized enzyme was left to sediment, the buffer was removed by pipetting, and the immobilized enzyme was used directly in biotransformations. Immobilization yield is reported in Table S2.

Immobilization on Purolite resin. 300 mg of Purolite resin pre-loaded with Co^{2+} were washed with KPi buffer (2 x 0.6 ml, 100 mM, pH 7.5). The buffer was removed after 5 minutes of manual shaking. Short pulse centrifugation was used to sediment the resin and the buffer was removed by pipetting. Purified LE-AmDH-v1 (335 µl from a 572 µM stock solution equal to 192 nmol, ca. 8.5 mg) and Cb-FDH (26 µl from a 1.33 mM stock solution equal to 35 nM, ca. 1.5 mg) were diluted with KPi buffer (100 mM, pH 7.5) and added to the resins (1.2 ml final volume). The vials were shaken for 2 h at 4 °C using an orbital shaker (90 rpm, horizontal positioning of vials). Small aliquots from the aqueous phase (30 µl) were taken before and after the immobilization procedure; their concentrations were determined using Bradford and visualized via SDS-PAGE. Immobilized enzyme was washed with KPi buffer (2 x 0.6 ml) and then incubated for 45 min in KPi buffer (1 x 1.2 ml). Finally, another washing step was performed with KPi buffer (2 x 0.6 ml) and the immobilized enzyme was ready for activity testing. Immobilization yield is reported in Table S2.

Immobilization on Sepabeads EC-EP/S, Relizyme 113/S and 403/S. Epoxy-functionalized Sepabeads EC-EP/S, Relizyme 113/S and 403/S (100 mg, each) were suspended in 5 ml KPi (100 mM, pH 7.5) in three different tubes. Purified LE-AmDH-v1 (335 µl from a 572 µM stock solution equal to 192 nmol, ca. 8.5 mg) and Cb-FDH (26 µl from a 1.33 mM stock solution equal to 35 nM, ca. 1.5 mg) were added in each tube (total enzyme ca. 10 mg, equal to 10% w w^{-1}, enzyme loading to support material).

The mixture was shaken on an orbital shaker at 4 °C for 72 h (90 rpm, mild shaking). After centrifugation (10.0 krpm, 1 min, 4 °C), the buffer solution was discarded and the beads were washed with KPi (100 mM, pH 7.5, 1 ml) for several minutes (600 rpm). After centrifugation, the remaining solution was discarded and the beads were washed with KPi (100 mM, pH 7.5, 1 ml) for 30 minutes (600 rpm). After centrifugation the supernatant was discarded, and beads were directly used in the activity measurements. Small aliquots from the aqueous phase (30 µl) were taken before and after the immobilization procedure and during the washing steps; their concentrations were determined using Bradford and visualized via SDS-PAGE. Immobilization yield is reported in Table S2.

For all immobilization procedures, SDS-PAGE analysis is shown in Fig. S3. Data on process for Immobilization are reported in Table S2.

Table S2. Immobilization yield for the co-immobilization of LE-AmDH-v1 and Cb-FDH on different carrier materials determined by Bradford assays.

Carrier materials	Total protein [mg]	Sample volume [ml]	Intended concentration [mg ml^{-1}]	Sample concentration before immobilization [mg ml^{-1}]	Sample concentration after immobilization [mg ml^{-1}]	Immobilization yield [%]	Absolute amount of enzyme per 100 mg of beads [mg]
EziG[1] (Opal)	10	10	1	0.82	0.46	44	4.4
Purolite	10	2.5	4	3.26	0.61	81	8.1
Sepabeads EC-EP/s	10	5.36	1.86	1.88	1.39	26	2.6
Relyzime 113/s	10	5.36	1.86	1.92	1.49	22	2.2
Relyzime 403/s	10	5.36	1.86	1.04	0.91	13	1.3

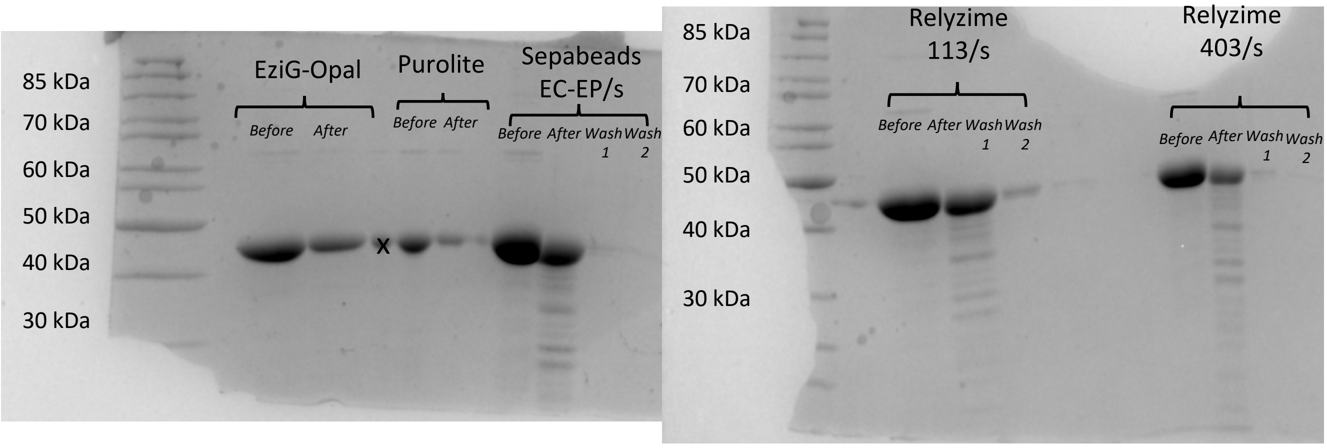

Fig. S3. Representative SDS-PAGE gels of pre- and after co-immobilization experiments with different carrier materials. Protein ladder: PageRuler™ Unstained Protein Ladder (ThermoFisher Scientific). Coomasie blue staining method.

Reaction conditions for the reductive amination of benzaldehyde (1): 1 ml final volume in 2 ml Eppendorf tubes; ammonia/ammonium formate buffer (750 mM, pH 8); NAD^+ (1 mM); **1** (20 mM) as substrate. Co-immobilized LE-AmDH-v1 and Cb-FDH prepared as reported above (ca. 25 mg of total mass enzyme plus beads for each sample). The biotransformations were incubated at 170 rpm and 30 °C in an orbital shaker for 24 h. Next, an aliquot of 500 µl of the reaction mixture was stopped with 70 µl of 10 M KOH, extracted once with 650 µl of EtOAc containing 10 mM toluene as internal standard (1 min vortexing + 5 min centrifugation, 4 °C, 14800 rpm) and dried over $MgSO_4$. Analytical yields (Table S3) were determined by GC-FID (see paragraph Analytics) using toluene as internal standard. Reactions were performed in duplicate.

Results of activity tests in batch experiments are reported in Table S3 and Fig. S4.

Table S3. Activity tests of co-immobilized LE-AmDH-v1 and Cb-FDH on different beads. Conditions: ammonia/ammonium formate buffer (750 mM pH 8), NAD^+ (1 mM), **1a** (20 mM), ca. 25 mg of beads. t = 24 h, T = 40 °C, 170 rpm.

Carrier materials	Analytical Yield **2** [%]
EziG - Opal	96 ± 8
Purolite	> 99
Sepabeads EC-EP/s	11
Relyzime 113/s	15 ± 7
Relyzime 403/s	2 ± 5

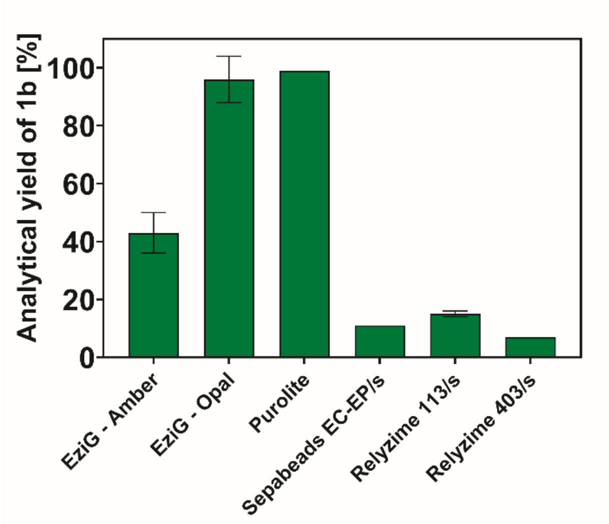

Fig. S4. Activity of LE-AmDH-v1 plus Cb-FDH co-immobilized on different supports at 750 mM ammonium/ammonia species concentration and 20 mM of **1**.

3.3. In-flow co-immobilization on EziG-Amber beads from cell free extract and initial experiments in flow

The bacterial cell pellet (1 g) containing overexpressed LE-AmDH-v1 or Cb-FDH were thawed and re-suspended in 0.9% Tris-HCl (100 mM, pH 7.8, 5 ml) and subsequently sonicated (5 min on time, 10 s on, 10 s off, 45% amp). Afterwards, samples were centrifuged (10 min, 4 °C, 14800 rpm) and the supernatant was filtered (0.45 µm filter pores).

A stainless-steel column (50 mm length x 2 mm diameter) was filled with EziG[3] (Fe-Amber) (500 mg) and hydrated with Tris-HCl buffer (50 ml, 100 mM, pH 7.8, flow 0.5 ml min^{-1}). The soluble protein fraction obtained after centrifugation of the CFE (ca. 100 mg total enzymes, 20 % w w^{-1}), prepared as reported in first paragraph of section 3.1., was loaded onto the column using a peristaltic pump (flow rate = 150 µl min^{-1}). After complete loading, the flow was stopped, and the crude cell lysate was left to incubate in the column for 45 min at room temperature. Next, Tris-HCl buffer (50 ml, 100 mM, pH 7.8, flow 0.5 ml min^{-1}) was flowed through the column to wash out the other protein impurities. Samples (20 µl) of the loaded CFE and the flow-through obtained during washing of the column were collected and analyzed by SDS-PAGE.

The column containing the co-immobilized enzymes was subsequently mounted on a Dionex P680 HPLC pump unit and conditioned by flowing the reaction buffer (ammonia/ammonium formate 30 ml, 750 mM, pH 8, flow rate 0.3 ml min^{-1}). After conditioning, the flow reactor containing the immobilized enzymes was disconnected from the HPLC line. Next, the reaction mixture (5 ml final volume; benzaldehyde (**1**) (20 mM) in ammonia/ammoniun formate buffer (750 mM, pH 8); NAD^+ (1 mM)) was injected into the system directly from the HPLC solvent lines at 1 ml min^{-1}. When all of the reagent's solution was injected, the input of the HPLC line was switched back to the reservoir containing the reaction buffer. At this stage, the flow rate was kept constant at 1 ml min^{-1} until all the void volume was flashed out (ca. 13 ml). Subsequently, the HPLC line was connected again to the column containing co-immobilized enzymes; the flow rate was set at 0.01 ml min^{-1}. Column was warmed up at 50 °C using a heated water bath, and the system was maintained at constant pressure through a BPR element (40 psi). The reactor outcome was collected in 2 ml Eppendorf tubes (ca. 1 ml in each tube).

Analysis of the fractions was performed as follows: The reaction was treated with 70 µl of 10 M KOH, extracted once with 650 µl of EtOAc containing 10 mM toluene as internal standard (1 min vortexing + 5 min centrifugation, 4 °C, 14800 rpm) and dried over $MgSO_4$. Analytical yields (Table S4) were determined by GC-FID (see paragraph Analytics).

Table S4. Reductive amination of **1** using LE-AmDH-v1 and Cb-FDH after in-flow co-immobilization from CFE. Conditions: ammonia/ammonium formate buffer (750 mM, pH 8), NAD^+ (1 mM) and **1** (20 mM); LE-AmDH-v1 and Cb-FDH were co-immobilized on EziG[3] (ca. 100 mg total enzymes, 20 % w w^{-1}, enzymes, carrier), flow rate = 0.01 ml min^{-1}, t = 400 min, T = 50 °C.

Fraction [min]	Recovered **1** [mM]	Recovered **2** [mM]
250 – 300	0	0
300 – 350	0	1.7
350 - 400	0	3.2

3.4. Co-immobilization of LE-AmDH-v1 and Cb-FDH on EziG[1] (Opal) beads at different enzyme loadings

3 vials containing 10 ± 0.2 mg of EziG[1] (Opal) carrier material was suspended in the immobilization buffer (Tris-HCl, 3x1 ml, 100 mM, pH 7.8). Purified LE-AmDH-v1 and Cb-FDH were added to the suspensions as follows:

- ca. 0.5 mg total enzymes, equal to 5 % w w^{-1}, enzyme loading to support material (16.8 µl of LE-AmDH-v1 from a 572 µM stock solution equal to 9.6 nmol, ca. 0.43 mg; 1.3 µl of Cb-FDH from a 1.33 mM stock solution equal to 1.7 nmol, ca. 0.07 mg)

- ca. 1 mg total enzymes, equal to 10 % w w^{-1}, enzyme loading to support material (34 µl of LE-AmDH-v1 from a 572 µM stock solution equal to 19 nmol, ca. 0.87 mg; 2.6 µl of Cb-FDH from a 1.33 mM stock solution equal to 3.5 nmol, ca. 0.15 mg)

- ca. 1.5 mg total enzymes, equal to 15 % w w^{-1}, enzyme loading to support (50 µl of LE-AmDH-v1 from a 572 µM stock solution equal to 29 nmol, ca. 1.27 mg; 3.9 µl of Cb-FDH from a 1.33 mM stock solution equal to 5.2 nmol, ca. 0.22 mg)

Mixtures were shaken on an orbital shaker (120 rpm) for 4 h at 4 °C. Small aliquots from the aqueous phase (20 µl) were taken before and after 30 min, 1 h, 2 h, 3 h and 4 h; their concentrations (Table S5) were determined using Bradford and visualized with SDS-PAGE (Fig. S5).

Table S5. Analysis via Bradford assays for the co-immobilization of LE-AmDH-v1 and Cb-FDH on EziG (Opal) with different enzymes loading on carrier material.

Enzyme loading [% w w^{-1}]	Sample concentration before immobilization [mg ml^{-1}]	After 30 min [mg ml^{-1}]	After 1 h [mg ml^{-1}]	After 2 h [mg ml^{-1}]	After 3 h [mg ml^{-1}]	After 4 h [mg ml^{-1}]
5	0.38	< 0.1	< 0.1	< 0.1	< 0.1	< 0.1
10	0.79	< 0.1	< 0.1	< 0.1	< 0.1	< 0.1
15	1.22	0.23	0.13	< 0.1	< 0.1	< 0.1

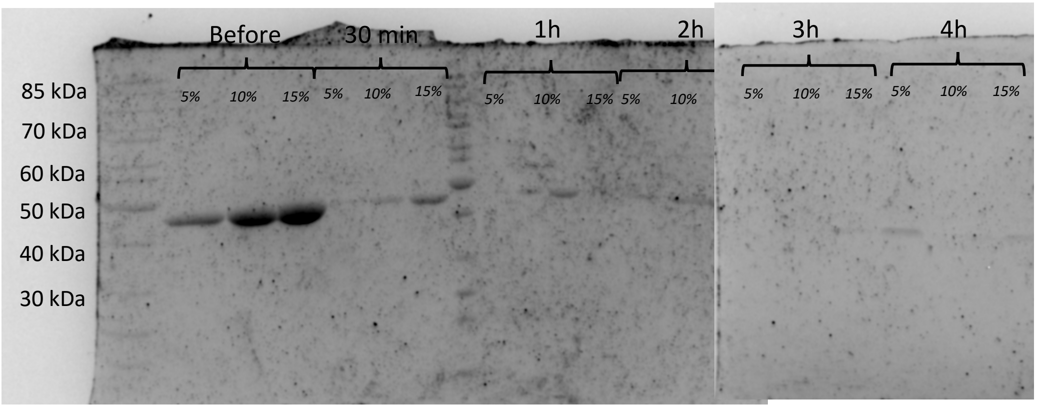

Fig. S5. SDS-PAGE gel of solution pre- and after co-immobilization of LE-AmDH-v1 and Cb-FDH on EziG[1] (Opal) carrier material. Protein ladder: PageRuler™ Unstained Protein Ladder (ThermoFisher Scientific). Coomasie blue staining method.

3.5. Co-immobilization of LE-AmDH-v1 and Cb-FDH from cell free extract and activity test

A 15 ml Falcon tube containing 100 mg of EziG[1] (Opal) carrier material was cooled down in an ice bath and suspended in the immobilization buffer (Tris-HCl, 10 ml, 100 mM, pH 7.8). Combined CFE, prepared as reported in section 3.1, containing LE-AmDH-v1 and Cb-FDH (total enzymes ca. 10 mg, equal to 10 % w w^{-1}, enzyme loading to support material) were added to the suspension and the mixture was shaken with an orbital shaker (120 rpm) for 3 h at 4 °C. Small aliquots from the aqueous phase (30 µl) were taken before and after the immobilization procedure; their concentrations were determined using Bradford analysis and visualized via SDS-PAGE. The carrier bearing immobilized enzyme was left to sediment, the buffer was removed by pipetting, and the immobilized enzyme was used directly in biotransformations.

Reaction conditions: 1 ml final volume in 2 ml Eppendorf tubes; ammonia/ammonium formate buffer (750 mM, pH 8); NAD^+ (1 mM); benzaldehyde (10 mM) as substrate. Co-immobilized LE-AmDH-v1 and Cb-FDH were prepared as reported above (ca. 25 mg total mass of enzymes plus beads, in each sample). The biotransformations were incubated at 170 rpm and 30 °C on an orbital shaker for 24 h. Next, 500 µl of the reaction mixture was stopped with 70 µl of 10 M KOH, extracted once with 650 µl of EtOAc containing 10 mM toluene as internal standard (1 min vortexing + 5 min centrifugation, 4 °C, 14800 rpm) and dried over $MgSO_4$. Analytical yields (Table S6) were determined by GC-FID (see paragraph Analytics) using toluene as internal standard. Reactions were performed in duplicate.

Table S6. Activity tests of co-immobilized LE-AmDH-v1 and Cb-FDH on EziG[1]. Conditions: ammonia/ammonium formate (750 mM, pH 8), NAD^+ (1 mM), **1 (**10 mM), ca. 25 mg of beads. t = 24 h, T = 40°C, 170 rpm.

Sample	Analytical Yield **2** [%]
Blank	0
LE-AmDH-v1 + Cb-FDH on EziG[1]	56 ± 6

3.6. In-flow co-immobilization of LE-AmDH-v1 and Cb-FDH from cell free extract

A stainless-steel column (50 mm length x 2 mm diameter) was filled with EziG[1] Fe-Opal (500 mg) and hydrated with Tris-HCl buffer (40 ml, 100 mM, pH 7.8, flow 0.5 ml min^{-1}). The soluble protein fraction of CFE prepared as reported in section 3.1 was loaded onto the column using a peristaltic pump (flow rate = 200 µl min^{-1}). After complete loading, the flow was stopped, and the cell lysate was left to incubate in the column for 30 min at room temperature. Then, Tris-HCl buffer (50 ml, 100 mM, pH 7.8, flow 0.5 ml min^{-1}) was flowed through the column to wash out any possibly unbound component. Buffer samples (20 µl) of the loading enzyme solution and of the flow-through obtained during washing were taken and immobilization was visualized via SDS-PAGE (Fig. S6).

The reaction was performed as reported in section 3.3 with following changes – column was conditioned with 20 ml of the buffer and th flow rate of the reaction was 0.02 ml min^{-1}.

Table S7. Reductive amination of benzaldehyde using LE-AmDH-v1 and Cb-FDH after in flow co-immobilization from CFE. Conditions: ammonia/ammonium formate (750 mM, pH 8), NAD^+ (1mM) and substrate **1** (20 mM); LE-AmDH-v1 and Cb-FDH were co-immobilized on EziG[3-] Fe -Amber beads, flow rate = 0.02 ml min^{-1}, t = 300 min, T = 50 °C.

Fraction [min]	Recovered **1** [mM]	Recovered **2** [mM]
100 – 150	1.4	1.4
150 – 200	4.3	3.1
200 – 250	7.4	3.5
250 - 300	7.9	3.3

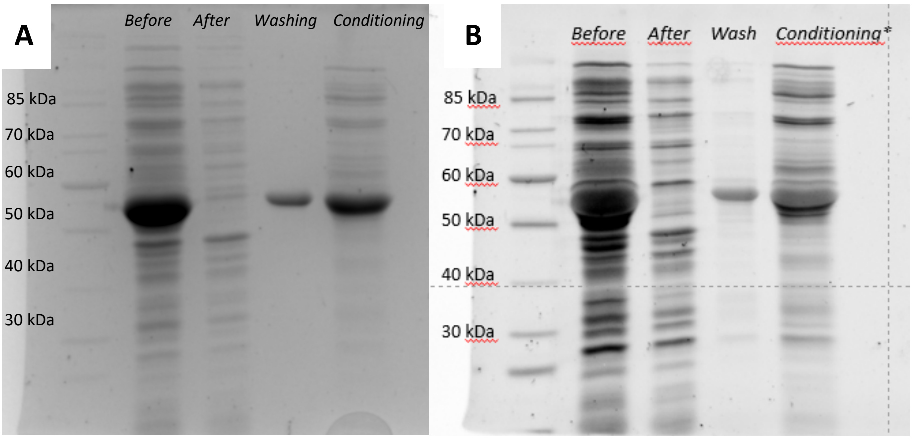

Fig. S6. SDS-PAGE gel of pre- and after in-flow co-immobilization of LE-AmDH-v1 and Cb-FDH on EziG[1] (Opal) carrier material from CFE. Protein ladder: PageRuler™ Unstained Protein Ladder (ThermoFisher Scientific). A) Coomasie blue staining method. B) UV detection.

Note: LE-AmDH-v1 cannot be visualized on SDS-PAGE via UV detection (B) because it does not have any L-trypthophan residue in its primary sequence. LE-AmDH-v1 can only be visualized by Coomassie staining (A). Therefore, the UV-detection (B) visualizes only the presence of Cb-FDH, whereas the Coomassie staining (A) visualizes both Cb-FDH and LE-AmDH-v1.

3.7. In-flow co-immobilization and cross-linking of dehydrogenase enzymes.

A stainless-steel column (50 mm length x 2 mm diameter) was filled with EziG[1] (Fe-Opal) (500 mg) and hydrated with Tris-HCl buffer (30 ml, 100 mM, pH 7.8, flow 0.5 ml min^{-1}). The soluble protein fraction of CFE prepared as reported in section 3.1 was loaded onto the column using a peristaltic pump (flow rate = 150 µl min^{-1}). After complete loading, the flow was stopped, and the cell lysate was left to incubate in the column for 30 min at r.t. Subsequently, a glutaraldehyde solution (5 % in KPi buffer, 10 mM, pH 7.6, final volume 5 ml) was flowed onto the column (0.3 ml min^{-1}) and then the column was washed with Tris-HCl buffer (100 mM, pH 8, 20 ml, 0.3 ml min^{-1}) in order to stop cross-linking reaction and to wash any unbound component. Buffer samples (20 µl) of the loading enzyme solution and of the flow-through obtained during washing were taken and immobilization was monitored through SDS-PAGE (Fig. S7).

The reaction was performed as reported in section 3.6.

Table S8. Reductive Amination of Benzaldehyde using LE-AmDH-v1 and Cb-FDH after in flow co-immobilization from CFE and attempted cross-linking using glutaraldehyde. Conditions: Ammonium formate 750 mM pH 8, NAD^+ 1mM and substrate 10 mM; co-immobilized LE-AmDH-v1 and Cb-FDH on EziG^{3-} Fe -Amber beads, flow rate = 0.02 ml min^{-1}, t = 300 min, T = 50 °C.

Fraction [min]	Recovered **1** [mM]	Recovered **2** [mM]
100 – 150	0.5	0
150 – 200	2.5	0
200 – 250	4.2	0
250 - 300	5.2	0

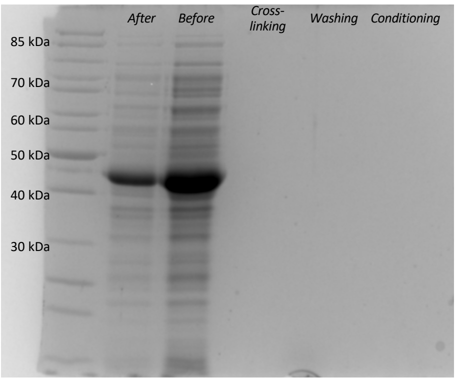

Fig. S7. SDS-PAGE gel of pre- and after in-flow co-immobilization of LE-AmDH-v1 and Cb-FDH EziG[1] – Opal carrier material from CFE and following cross-lonking. Protein ladder: PageRuler™ Unstained Protein Ladder (ThermoFisher Scientific). Coomasie blue staining method.

4. Co-entrampment of LE-AmDH-v1 and Cb-FDH on an agarose-based flow reactor

4.1. Preparation of agarose-based hydrogel "bricks" for activity tests

An agarose solution (3% w w^{-1}, + NaCl 10 mM, 2 ml final volume) was prepared in Tris-HCl buffer (100 mM, pH 7.8) and heated up with a microwave (300 W, 10 sec) until a clear solution was obtained. Afterwards, the solution was allowed to cool for up to 2-3 minutes and then purified LE-AmDH-v1 (90 µM as final concentration) and Cb-FDH (16 µM as final concentration) were added. Quickly the resulting mixture was poured into a hole of an Eppendorf tube holder and allowed to cool down and solidify. Finally, the "solid agarose brick" was removed from the mold, split in two equal parts and directly used for biotransformation (Fig. S8).

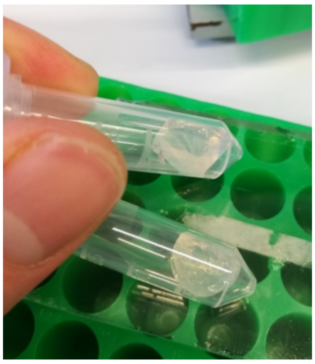

Fig. S8. Molded hydrogel brick ready for biotransformation.

4.2. Preliminary activity test in batch

Agarose-based hydrogel bricks containing entrapped LE-AmDH-v1 and Cb-FDH (90 µM and 16 µM) were prepared as reported in section 4.1.

Reaction conditions: 1 ml final volume in 2 ml Eppendorf tubes; ammonia/ammonium formate buffer (750 mM, pH 8); NAD^+ (1mM); benzaldehyde (10 mM) as substrate. Entrapped LE-AmDH-v1 and Cb-FDH were prepared as reported above. The biotransformations were incubated at 170 rpm and 40 °C in an orbital shaker for 24 h. Next, an alquot of 500 µl of the reaction mixture was stopped with 70 µl of 10 M KOH, extracted once with 650 µl of EtOAc containing 10 mM toluene as internal standard (1 min vortexing + 5 min centrifugation, 4 °C, 14800 rpm) and dried over $MgSO_4$. Furthermore, the second half of the reaction mixture (500 µl) containing also the agarose-based hydrogel brick were extracted with the same procedure described above. Analytical yields (Table S9) were determined by GC-FID (see paragraph Analytics) using toluene as internal standard. Reactions were performed in duplicate.

Table S9. Testing of entrapped LE-AmDH-v1 and Cb-FDH in agarose-based hydrogel. Conditions: ammonia/ammonium formate (750 mM, pH 8), NAD^+ (1 mM), substrate (10 mM), 1 cm x 1 cm gel brick with LE-AmDH-v1 (90 µM) and FDH (16 µM). t = 24 h, T = 40 °C and 170 rpm.

Extracted	Recovered **2** [mM]
Reaction mixture	6.9
Reaction mixture + agarose hydrogel (reactor)	9.3

4.3. Recycling test

Agarose-based hydrogel bricks containing entrapped LE-AmDH-v1 and Cb-FDH (90 µM and 16 µM) were prepared as reported in section 4.1.

Reaction conditions: 1 ml final volume in 2 ml Eppendorf tubes; ammonia/ammonium formate buffer (750 mM, pH 8); NAD^+ (1 mM); benzaldehyde (10 mM) as substrate. Entrapped LE-AmDH-v1 and Cb-FDH were prepared as reported above. The biotransformations were incubated at 170 rpm and 40 °C in an orbital shaker for 24 h. Next, an aliquot of 500 µl of the

reaction mixture was stopped with 70 µl of 10 M KOH, extracted once with 650 µl of EtOAc containing 10 mM toluene as internal standard (1 min vortexing + 5 min centrifugation, 4 °C, 14800 rpm) and dried over $MgSO_4$.

The agarose-based hydrogel brick was then removed from the Eppendorf tube, transferred into a new one and incubated again for 24 h (this procedure was repeated for 7 days). Between the 3rd and the 4th cycle, agarose-based hydrogel bricks were left over weekend in the fridge, hydrated with substrate-free reaction buffer. Finally, in the last cycle, also the second half of the reaction mixture (500 µl) containing the agarose-based hydrogel brick was extracted with the same procedure described above. Analytical yields (Table S10) were determined by GC-FID (see paragraph Analytics) using toluene as internal standard. Reactions were performed in duplicate.

Table S10. Recycling test of entrapped LE-AmDH-v1 and Cb-FDH in agarose-based hydrogel. Conditions: ammonia/ammonium formate (750 mM pH 8), NAD^+ (1 mM), substrate (10 mM), 1 cm x 1 cm gel brick with LE-AmDH-v1 (90 µM) and FDH (16 µM). t = 24 h, T = 40 °C and 170 rpm.

Cycle	Overall age of the reactor [day]	Recovered **1** [mM]	Recovered **2** [mM]
1	New	0	4.9 ± 1.3
2	1	0.04 ± 0.04	> 9.9
3	2	0.33 ± 0.01	8.2 ± 0.7
4	3	1.1 ± 0.1	5.4 ± 0.7
5	6	3.8 ± 0.1	2.5 ± 0.6
6	7	5.6 ± 0.3	1.0 ± 0.4
7	8	6.3 ± 0.4	1.3 ± 0.2

4.4. In-flow reductive amination using entrapped LE-AmDH-v1 and Cb-FDH using an agarose-based hydrogel flow reactor

An agarose-based flow reactor (Type II-v1) containing entrapped LE-AmDH-v1 and Cb-FDH (90 µM and 16 µM) were prepared as reported in the general procedure (main manuscript, experimental part). This led us to obtain a longer reactor 1 cm x 6 cm (diameter x length) with several channels inside. After removing the solidified reactor from the mold, this was entered inside an empty HPLC-column (1 cm x 10 cm, diameter x length), and the assembled reactor (Fig S9) was manually filled with reaction buffer (ammonia/ammonium formate, pH 8, 750 mM).

The assembled flow reactor was subsequently mounted on a KD Scientific KD 100 syringe pump unit equipped with a Terumo plastic syringe (10 ml) containing the reaction mixture (10 ml). The flow rate was set at 0.02 ml min^{-1} and the column was heated up (50 °C) by using a warm water bath. Reactor outcome was collected in 15 ml Falcon tubes. Afterwards, 500 µl of the outflow were mixed with 70 µl of 10 M KOH, extracted once with 650 µl of EtOAc containing 10 mM toluene as internal standard (1 min vortexing + 5 min centrifugation, 4 °C, 14800 rpm) and dried over $MgSO_4$. Analytical yields were determined by GC-FID (see paragraph Analytics) using toluene as internal standard.

Reaction mixture: 10 ml final volume; benzaldehyde (10 mM) in ammonia/ammoniun formate buffer (750 mM, pH 8); NAD^+ (1 mM).

Results. For each collected fraction: 5,1 % residual starting material; 95,8 % product analytical yield.

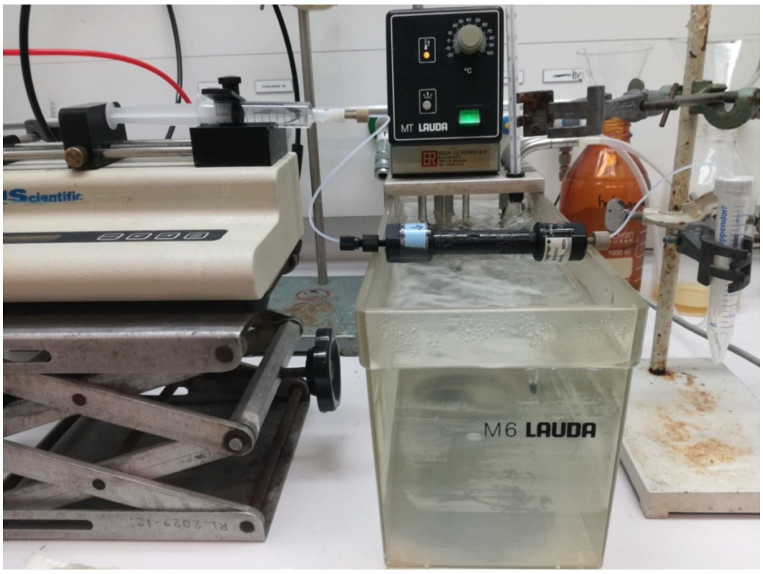

Fig. S9. Flow setup used for the in-flow biotransformation.

4.5. Continuous-flow reductive amination using entrapped LE-AmDH-v1 and Cb-FDH in an agarose-based hydrogel flow reactor

An agarose-based flow reactor (Type II-v2) containing entrapped LE-AmDH-v1 and Cb-FDH (90 µM and 16 µM) were prepared as reported in the general procedure (main manuscript, experimental part). After removing the solidified reactor from the mold, it was inserted into an empty HPLC-column (1 cm x 10 cm, diameter x length), and the assembled reactor was manually filled with reaction buffer (ammonia/ammonium formate, pH 8, 750 mM).

The reaction was performed according to the procedure described in section 3.6. Reactor outcome was collected in small round-bottom flasks for 24 h. 2× 500 µl aliquots of the outflow were mixed with 140 µl of 10 M KOH, extracted once with 650 µl of EtOAc containing 10 mM toluene as internal standard (1 min vortexing + 5 min centrifugation, 4 °C, 14800 rpm) and dried over $MgSO_4$. Analytical yields (Table S11) were determined by GC-FID (see paragraph Analytics) using toluene as internal standard.

Reaction mixture: 144 ml final volume; benzaldehyde (10 mM) in ammonia/ammoniun formate buffer (750 mM, pH 8); NAD^+ (1 mM).

Table S11. Reductive amination in continuous flow utilizing entrapped LE-AmDH-v1 and Cb-FDH in agarose-based hydrogel. Conditions: ammonia/ammonium formate (750 mM pH 8), NAD^+ (1 mM), substrate **1** (10 mM), 1 cm x 10 cm reactor containing LE-AmDH-v1 (90 µM) and Cb-FDH (16 µM). t = 24 h, T = 50 °C.

Sample	Recovered **1** [mM]	Recovered **2** [mM]
1	0	10.9
2	0	10.1
Average	0	10.5 ± 0.4

4.6. Continuous-flow reductive amination using entrapped LE-AmDH-v1 and Cb-FDH on an agarose-based hydrogel flow reactor at 30 mM substrate loading

Experimental details are reported in the main manuscript, experimental part. The process yielded 175.3 mg of product with 90% purity, thus resulting in 157.8 mg (1.47 mmol) of pure **2**. However, based on the volume of collected fractions and their concentration (determined using the internal standard, Table S12), the theoretical analytical yield of **2** was estimated to be higher, namley 220.5 mg (2.06 mmol, 47.8%). Threfore, the yield in the extraction step of volatile pure **2** was calculated to be 71.6% [(157.8 mg / 220.5 mg)*100].
In summary, the analytical yield of the flow reaction was 47.8%, while the final isolated yield (after extraction) was 34.2%. (based on 71.6% of yield in the extraction step and 47.8% of yield in the flow reaction.).

Table S12. Reductive amination in continuous flow utilizing entrapped LE-AmDH-v1 & Cb-FDH in agarose-based hydrogel. Conditions: ammonium formate 750 mM pH 8, NAD^+ 1 mM, substrate 30 mM, 1 cm x 10 cm reactor containing LE-AmDH-v1 (90 µM) and FDH (16 µM). t = 24h, t = 40°C and 170 rpm.

Cycle	Collection termination [h]	Recovered **1** [mM]	Recovered **2** [mM]
1	24	0.5	8.0
2	48	4.3	26.9
3	72	8.8	18.0
4	96	16.4	12.0
5	120	22.2	7.4
Wash	178	7.0	1.6

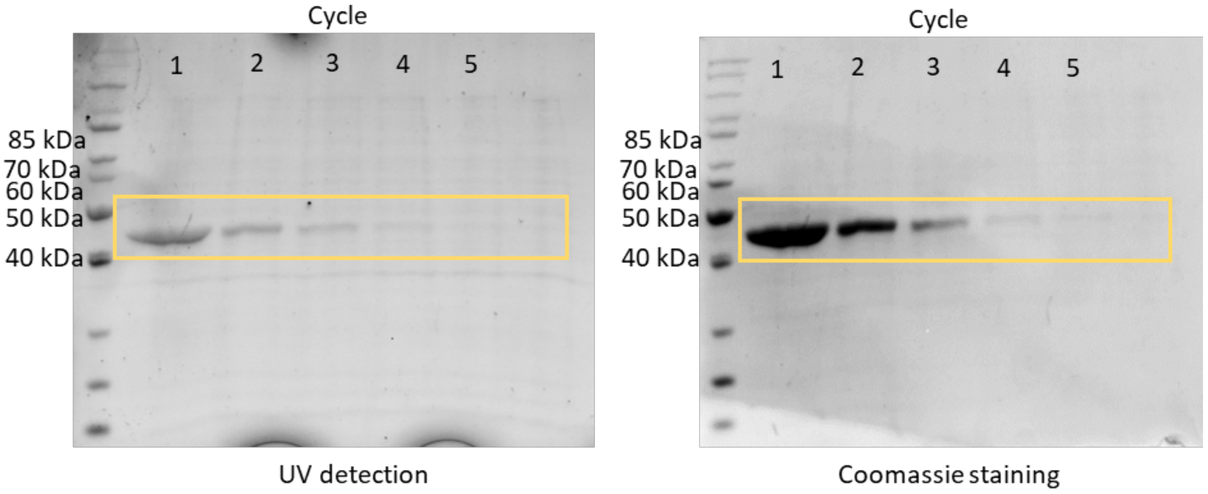

Fig. S10. SDS-PAGE to study the retention of LE-AmDH-v1 and Cb-FDH inside the agarose reactor. Protein ladder: PageRuler™ Unstained Protein Ladder (ThermoFisher Scientific). Coomasie blue staining method.

Note: LE-AmDH-v1 cannot be visualized on SDS-PAGE via UV detection (left) because it does not have any L-trypthophan residue in its primary sequence. LE-AmDH-v1 can only be visualized by Coomassie staining (right). Therefore, the UV-detection (left) visualizes only the presence of Cb-FDH, whereas the Coomassie staining (right) visualizes both Cb-FDH and LE-AmDH-v1. Since Cb-FDH is loaded at lower amount in the agarose gel, we conclude that Cb-FDH has a higher tendency than LE-AmDH-v1 to be eluted out of the agarose gel.

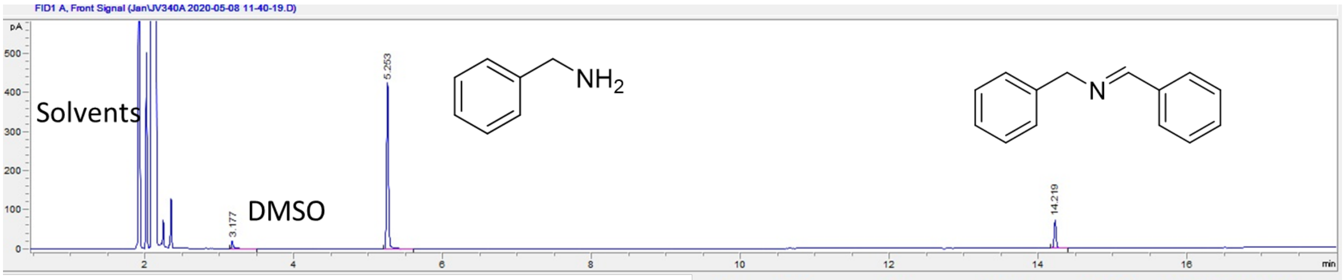

#	Time	Area	Height	Width	Area%	Symmetry
1	3.177	34.2	19.9	0.026	3.721	0.375
2	5.253	746.5	424.6	0.0275	81.181	0.917
3	14.219	138.8	72.2	0.0294	15.099	0.868

Fig. S11. Purity analysis of **2** obtained from long-term experiment.

Table S13. Reductive amination in continuous flow utilizing entrapped LE-AmDH-v1 and Cb-FDH in agarose-based hydrogel. Conditions: ammonia/ammonium formate (750 mM, pH 8), NAD+ (1 mM), substrate (30 mM), 1 cm × 10 cm reactor containing LE-AmDH-v1 (90 µM) and FDH (16 µM). t = 24 h, T = 40 °C and 170 rpm.

Cycle	Collection termination [h]	Fraction volume [ml]	Recovered **2** [mM]	Theoretical yield of **2** [mg]
1	24	27	8.0	23.0
2	48	27	26.9	77.7
3	72	28	18.0	54.0
4	96	24	12.0	31.0
5	120	29	7.4	23.1
Wash	178	70	1.6	11.7
Total	n.a.	205	n.a.	220.5

4.7. Determination of process parameters from optimized experiment

To characterize the flow process, we have used several parameters such as yield per gram of catalyst and space-time-yield. All parameters (including the isolated yields and the parameters determined earlier) are listed in Table 2, main manuscript. Additionally, the equations that were used for the calculation of the parameters (if not listed previously) are listed below. Analytical yield was used as a mass of product. Molar mass of LE-AmDH-v1 and Cb-FDH is 44569 and 42429 Da, respectively.[1] The volume of the reactor was calculated in section 4.2 and the reaction time when the flowing of the reaction mixture was stopped is listed in Table S12 (cycle 5).

Enzyme loading (grams) in the reactor ($m_{protein}$)

$$m_{protein} = V_{reactor} * (c_{LE-AmDH-v1} * M_{LE-AmDH-v1} + c_{CbFDH} * M_{CbFDH})$$
$$m_{protein} = 0.006 * (0.00009 * 44569 + 0.000016 * 42429)$$
$$m_{protein} = 0.0281\ g$$

Yield (grams) per gram of catalyst

$$yield\ per\ mass\ of\ catalyst = \frac{m_{product}}{m_{protein}}$$
$$yield\ per\ mass\ of\ catalyst = \frac{0.2205}{0.0281}$$
$$yield\ per\ mass\ of\ catalyst = 7.85\ g\ g^{-1}$$

Yield (moles) per mole of catalyst

$$yield\ per\ mole\ of\ catalyst = \frac{n_{product}}{(V_{reactor} * c_{LE-AmDH-v1}) + (V_{reactor} * c_{CbFDH})}$$
$$yield\ per\ mole\ of\ catalyst = \frac{0.00206}{(0.006 * 0.00009) + (0.006 * 0.000016)}$$
$$yield\ per\ mole\ of\ catalyst\ = 3239\ mol\ mol^{-1}$$

Space time yield (STY)

$$STY = \frac{m_{product}}{V_{reactor} * t_{reaction}}$$
$$STY = \frac{0.2205}{0.006 * 120}$$
$$STY = 0.31\ g\ l^{-1} h^{-1}$$

4.8. Oligomerization of LE-AmDH-v1

Oligomerization status of LE-AmDH-v1 was determined via size exclusion chromatography using AKTA (GE Healthcare) chromatography system equipped with Superdex 200 26/600 column using 20 mM Tris-HCl pH 8 buffer supplemented with 150 mM NaCl. The molecular weight was estimated using the set of molecular weight standards (29 kDa – 669 kDa, 25 mg ml^{-1}, Figure S12). Table S14 contains the outcome of the size exclusion analysis.

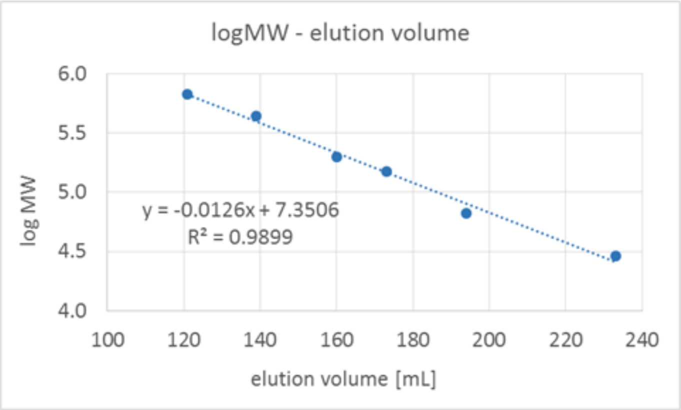

Fig. S12. SEC calibration curve for the determination of the molecular weight of LE-AmDH-v1

Table S14. Oligomerization state of LE-AmDH-v1 calculated using the SEC calibration curve.

Oligomerization status	elution volume [mL]
monomer	214
dimer	190
trimer	176
tetramer	166
hexamer	153
elution time	170
calculated MW	160.8 kDa
estimated oligomerization status	tetramer

5. Analytics

GC-FID was performed on an Agilent 7890B chromatograph using H_2 as carrier gas.

<u>Columns</u>: Agilent J&W HP-5 (30 m, 320 µm, 0.25 µm)

<u>Method</u>: T injector 250 °C; constant pressure 4.0 psi; temperature program: 60 °C, hold 0 min; 10 °C min^{-1} to 160 °C, hold 0 min; 20 °C min^{-1} to 280 °C, hold 2 min. Split ratio = 20:1

Retention times	- IS (toluene)	2.7 min
	- co-solvent (DMSO)	3.1 min
	- benzaldehyde 1	4.5 min
	- benzylamine 2	5.1 min

6. GC chromatograms

6.1. In-flow reductive amination using co-immobilized LE-AmDH-v1 and Cb-FDH on EziG³(Fe- Amber) beads

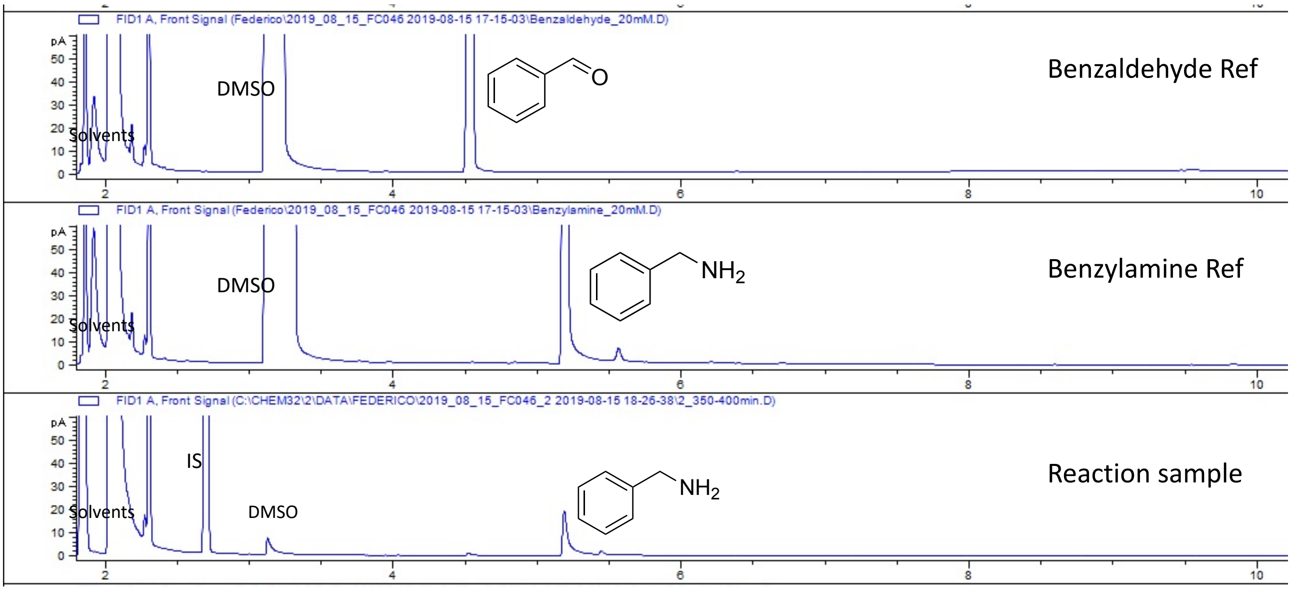

Fig. S13. In-flow reductive amination of benzaldehyde to benzylamine using co-immobilized LE-AmDH-v1 and Cb-FDH on EziG³ (Amber) beads. Solvents: solvent peak; IS: internal standard; DMSO: dimethylsulfoxide.

6.2. In-flow reductive amination using co-entrapped LE-AmDH-v1 and Cb-FDH on an agarose-based hydrogel flow reactor

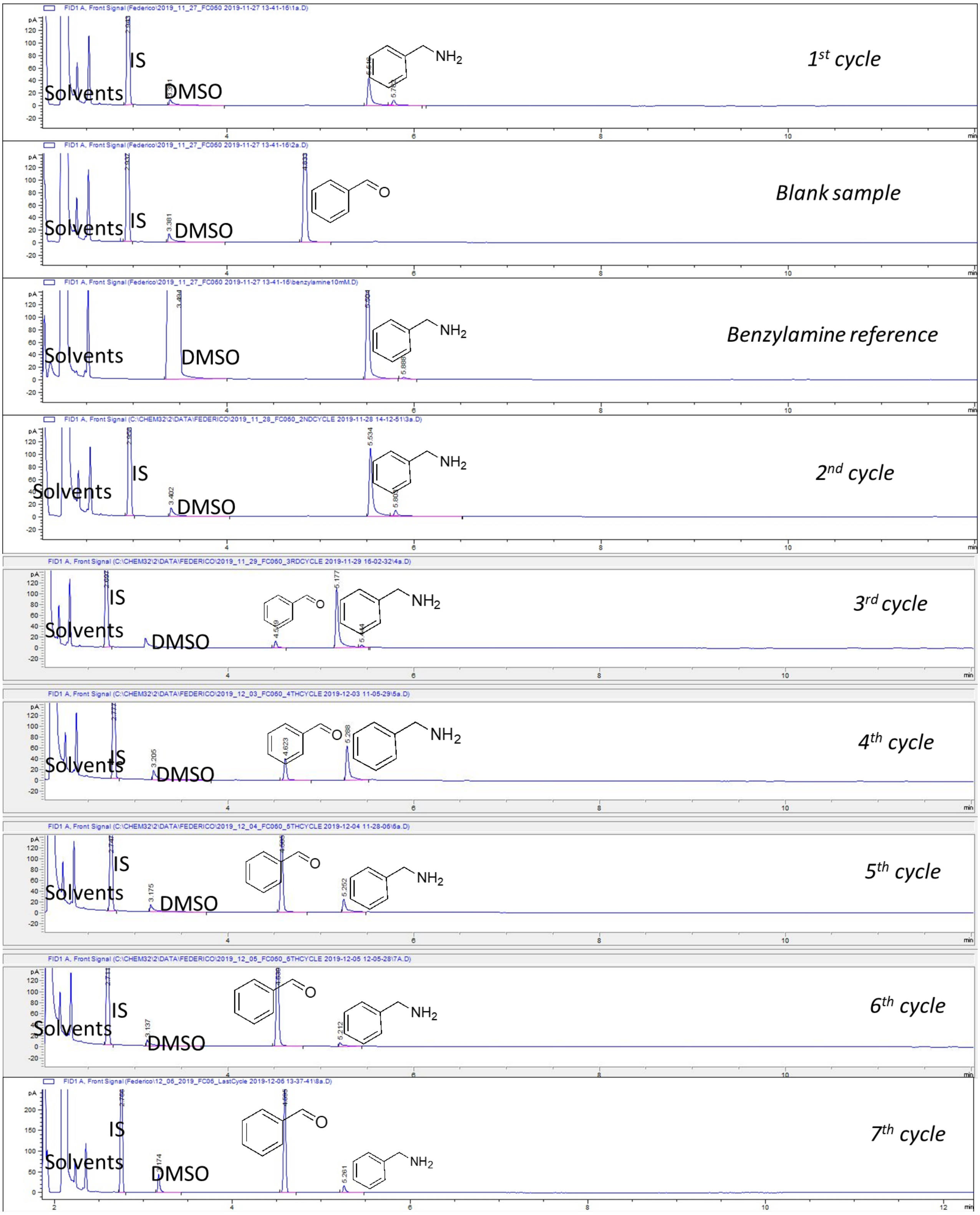

Fig. S14. Recycling test of agarose-based hydrogel reactor containing LE-AmDH-v1 and Cb-FDH. Solvents: solvent peak; IS: internal standard; DMSO: dimethylsulfoxide.

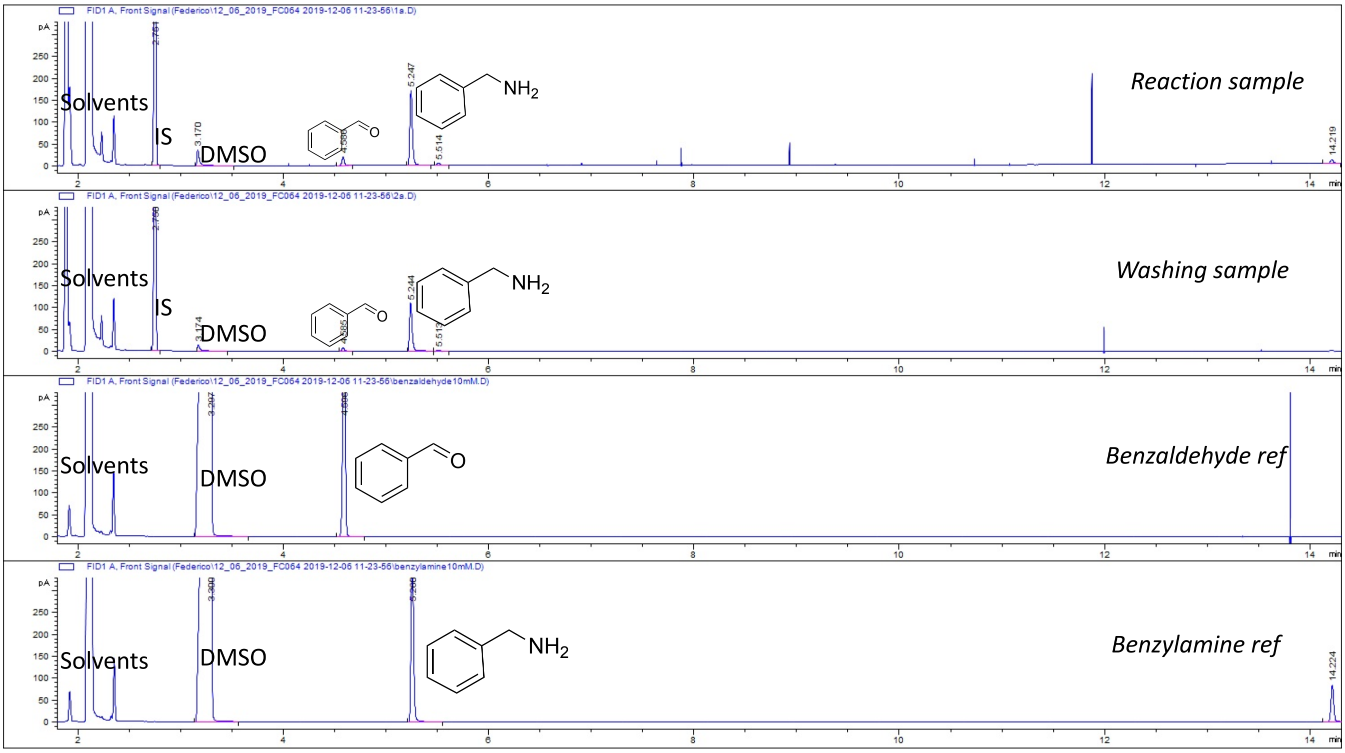

Fig. S15. In-flow reductive amination of benzaldehyde using an agarose-based hydrogel reactor containing LE-AmDH-v1 and Cb-FDH. Solvents: solvent peak; IS: internal standard; DMSO: dimethylsulfoxide.

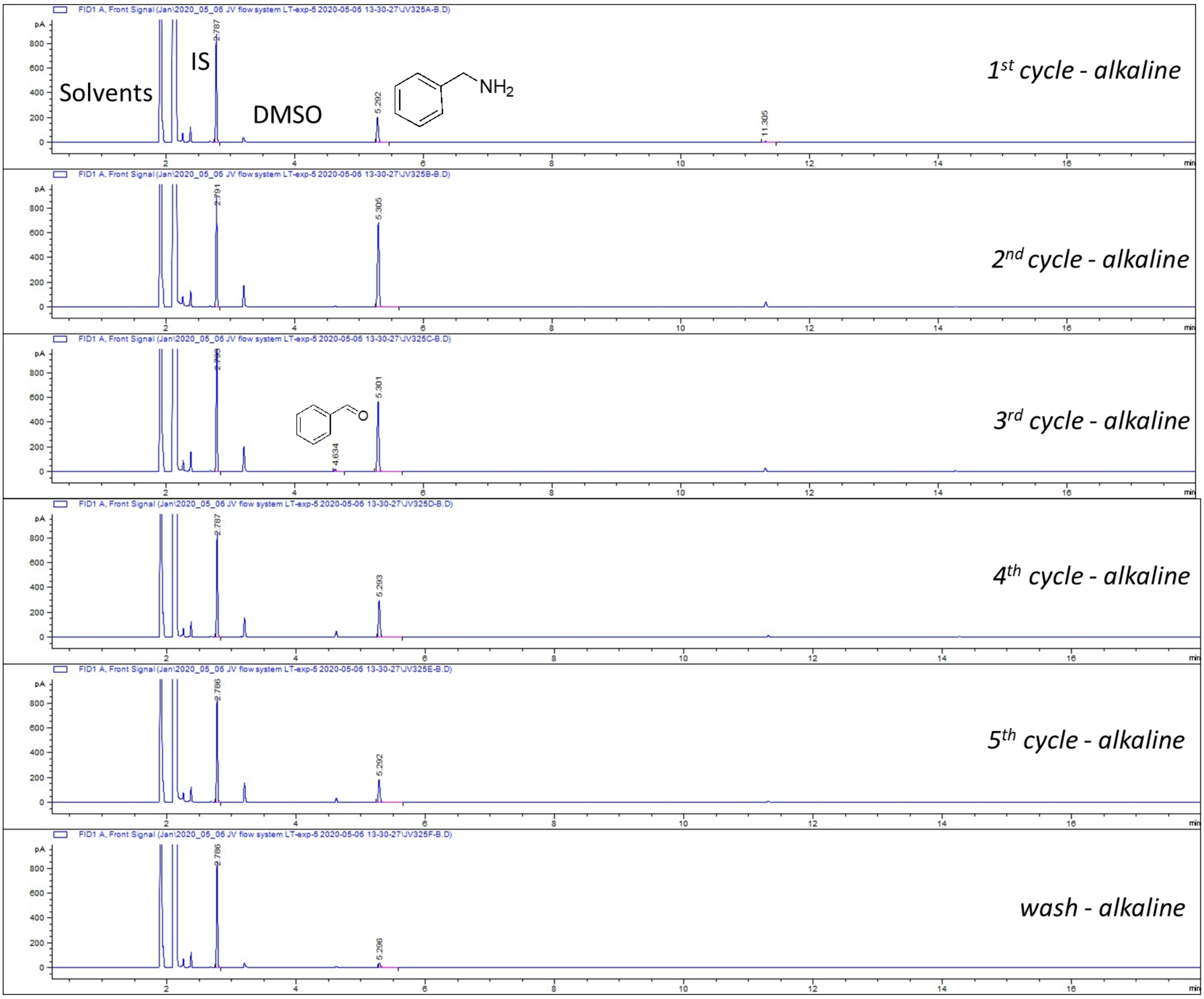

Fig. S16. In-flow reductive amination of benzaldehyde using an agarose-based hydrogel reactor containing LE-AmDH-v1 and Cb-FDH (long term experiment), alkaline extraction. Solvents: solvent peak; IS: internal standard; DMSO: dimethylsulfoxide.

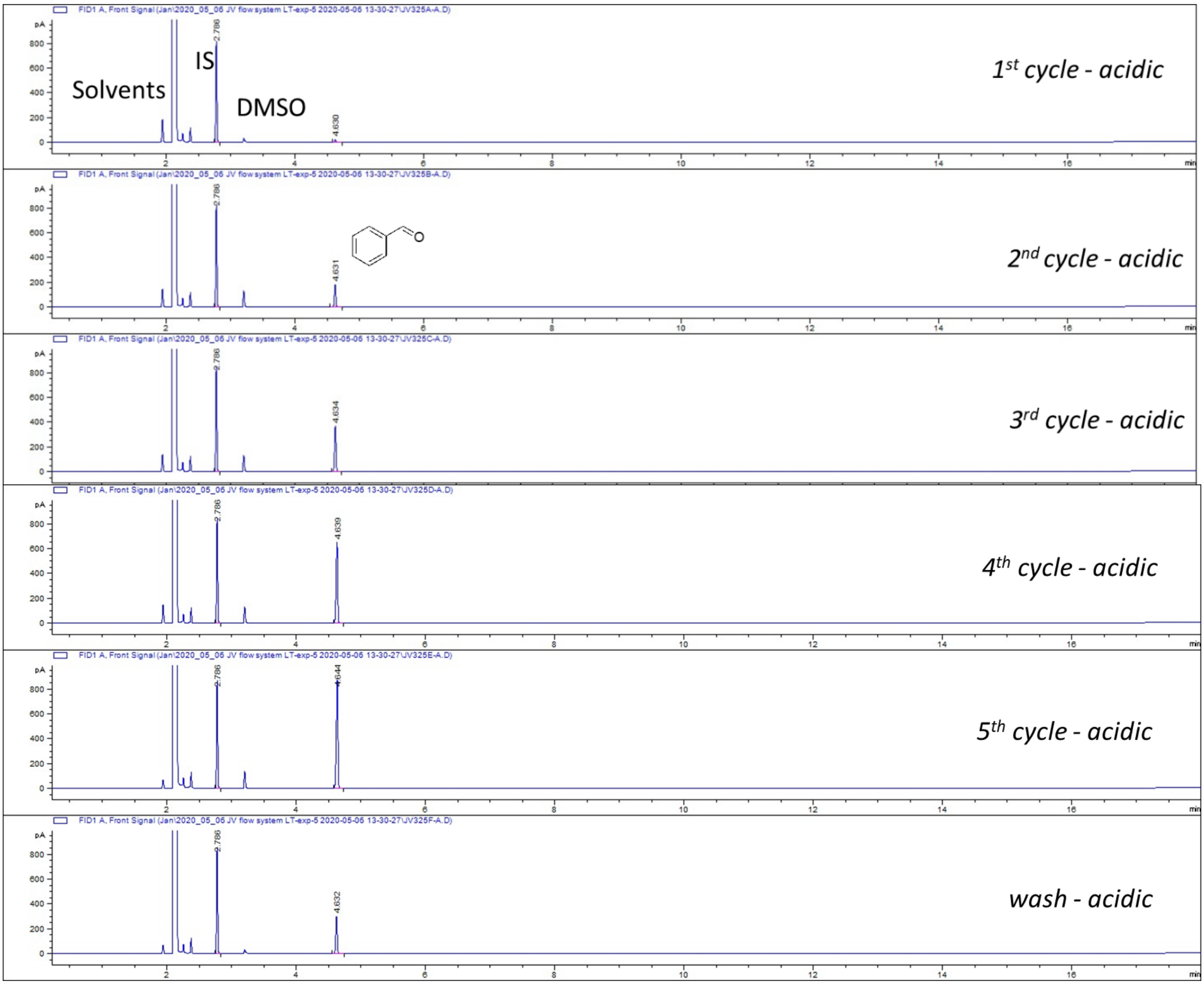

Fig. S17. In-flow reductive amination of benzaldehyde using an agarose-based hydrogel reactor containing LE-AmDH-v1 and Cb-FDH (long term experiment), acidic extraction. Solvents: solvent peak; IS: internal standard; DMSO: dimethylsulfoxide.

7. References

[1] V. Tseliou, T. Knaus, M. F. Masman, M. L. Corrado, F. G. Mutti, *Nature Communications* **2019**, *10*, 3717.
